# Mechanism of resonant electron emission from the deprotonated GFP chromophore and its biomimetics†
†Electronic supplementary information (ESI) available: Experimental and computational details, calculated VEEs in anions and radicals, orbitals involved in electronic transitions, detailed discussion of direct and resonant spectral shapes, mechanism of vibrational autodetachment out of S_1_ above the S_1_/D_0_ crossing, impact of the substituents, and coordinates of the optimized structures. See DOI: 10.1039/c6sc05529j


**DOI:** 10.1039/c6sc05529j

**Published:** 2017-02-06

**Authors:** Anastasia V. Bochenkova, Ciarán R. S. Mooney, Michael A. Parkes, Joanne L. Woodhouse, Lijuan Zhang, Ross Lewin, John M. Ward, Helen C. Hailes, Lars H. Andersen, Helen H. Fielding

**Affiliations:** a Department of Chemistry , Lomonosov Moscow State University , 119991 Moscow , Russia . Email: bochenkova@phys.chem.msu.ru; b Department of Chemistry , University College London , 20 Gordon Street , London WC1H 0AJ , UK . Email: h.h.fielding@ucl.ac.uk; c Department of Biochemical Engineering , UCL , Bernard Katz Building, Gordon Street , London , WC1E 0AH , UK; d Department of Physics and Astronomy , Aarhus University , DK-8000 Aarhus C , Denmark

## Abstract

The Green Fluorescent Protein (GFP), which is widely used in bioimaging, is known to undergo light-induced redox transformations. Electron transfer is thought to occur resonantly through excited states of its chromophore; however, a detailed understanding of the electron gateway states of the chromophore is still missing. Here, we use photoelectron spectroscopy and high-level quantum chemistry calculations to show that following UV excitation, the ultrafast electron dynamics in the chromophore anion proceeds *via* an excited shape resonance strongly coupled to the open continuum. The impact of this state is found across the entire 355–315 nm excitation range, from above the first bound–bound transition to below the opening of higher-lying continua. By disentangling the electron dynamics in the photodetachment channels, we provide an important reference for the adiabatic position of the electron gateway state, which is located at 348 nm, and discover the source of the curiously large widths of the photoelectron spectra that have been reported in the literature. By introducing chemical modifications to the GFP chromophore, we show that the detachment threshold and the position of the gateway state, and hence the underlying excited-state dynamics, can be changed systematically. This enables a fine tuning of the intrinsic electron emission properties of the GFP chromophore and has significant implications for its function, suggesting that the biomimetic GFP chromophores are more stable to photooxidation.

## Abstract 2

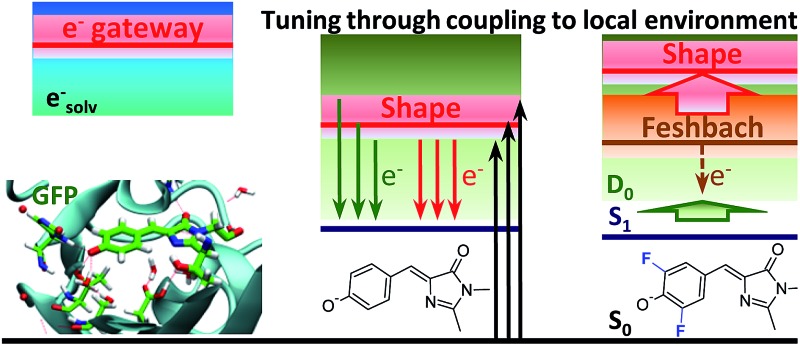
By uncovering the mechanism of UV resonant electron emission, we show that the GFP biomimetics are more stable to photooxidation.

## Introduction

**Fig. 1:**
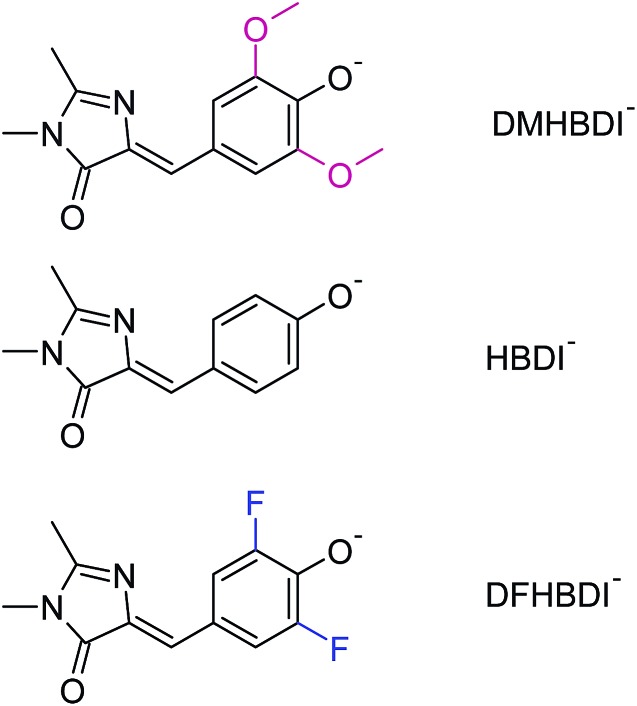
Native and biomimetic deprotonated GFP chromophores: 3,5-dimethoxy-4-hydroxybenzylidene-2,3-dimethylimidazolinone (DMHBDI), 4-hydroxybenzylidene-2,3-dimethylimidazolinone (HBDI), and 3,5-difluoro-4-hydroxybenzylidene-2,3-dimethylimidazolinone (DFHBDI).

Green fluorescent protein (GFP)^1^ and its derivatives have revolutionised biology through a diverse range of applications including advanced fluorescence imaging and biosensing.^2,3^ The molecular light machine at the heart of GFP is an extended π-system based on 4-hydroxybenzylidene-2,3-dimethylimidazolinone (HBDI) (Fig. 1) covalently bonded to the protein, which is wrapped around it with a β-barrel structure. The wild-type GFP chromophore is formed by a cyclisation reaction between three amino acid residues, S65, Y66 and G67, followed by an oxidation reaction. In its deprotonated anionic form, GFP is strongly fluorescent and has a quantum yield *Φ* = 0.79. The fluorescence is lost if the protein is denatured but returns upon renaturation or cooling below the glass-transition temperature.^4^ The isolated chromophore is barely fluorescent in the gas phase^5^ or in solution,^6^ but some close analogues have recently been shown to become strongly fluorescent when bound to selected ribonucleic acid (RNA) sequences^7,8^ or incorporated in rigid frameworks.^9^ The fluorescence wavelength of GFPs can be tuned by making mutations to the protein and, in this way, a whole palette of fluorescent proteins has been discovered and developed to cover the entire visible spectrum.^10^

Upon irradiation with UV and visible light, wild-type GFP and some GFP-like proteins undergo irreversible redox transformations,^11^ such as oxidative decarboxylation.^12–15^ Both neutral (phenol) and anionic (phenolate) forms of the chromophores, in various excited states, have been proposed to act as oxidants by accepting an electron from a nearby glutamate residue and initiating its decarboxylation though a so-called Kolbe-type mechanism.^12,13^ The thermal oxidative decarboxylation of carboxylic acids in solution, with, for example, manganese(iii) complexes acting as oxidants are well known in chemistry.^16^ However, the nature of the transient oxidant species formed in GFP and GFP-like proteins upon irradiation with light is not known, and there is no direct experimental evidence that electronically excited chromophores, regardless of the protonation state or the excitation wavelength, act as oxidants. The photo-transformation of wild-type GFP has been shown to be more efficient following excitation in the UV compared to the visible range of the electromagnetic spectrum.^13^ Furthermore, there is a wavelength-dependent threshold in laser power density for generating photoconverted GFP, and the decarboxylation can be turned off when visible light is used.^13^ It has also been shown that a consecutive two-photon absorption process is operative upon irradiation with visible light in a similar photoconversion of DsRed with the chromophore in its anionic form, and the importance of the photon flux in controlling the photoconversion process has been demonstrated.^14^ This indicates that the photoconversion occurs through higher electronically excited states of the chromophores, and the anionic form is directly involved in the reaction mechanism. Therefore, knowledge of the electron gateway states of the chromophore is required for establishing the detailed reaction mechanism and, ultimately, for controlling the photoconversion process.

Since the UV absorption of the chromophore overlaps heavily with the absorption of aromatic amino acid residues, the higher-lying excited states of the chromophore are not amenable to direct characterization in GFP. However, the excited states of HBDI^–^ can be probed in the gas phase by exploiting their resonant, metastable nature with respect to the open electron continuum. The pattern of excited states and their positions in the isolated chromophore provide an essential reference for those inside the protein.

Action-absorption^5,17,18^ and photoelectron^19–25^ spectroscopy have previously been used to study the electron emission properties of HBDI^–^ in the visible and UV regions. Using vibrationally resolved photoelectron spectroscopy, an adiabatic electron detachment threshold has been found at 2.73 eV.^22^ Photoelectron spectroscopy also allows us to obtain important information about the location of resonance states in the anion; however, broad and unresolved profiles and an interplay between direct and indirect (resonant) photodetachment (PD) channels make assignments difficult. Furthermore, internal conversion to lower-lying electronic states of the anion, as has recently been suggested,^24^ may also complicate the analysis of photoelectron spectra. The most prominent feature of HBDI^–^ photoelectron spectra measured within a 355–315 nm range of excitation wavelength is a considerable increase of the widths of the photoelectron distributions.^17,23,24^ The origin of this broadening is still being debated.^23,24^ An accurate theoretical description of these complex spectra is therefore crucial for unraveling the role of the excited states in the electron emission properties of the GFP chromophore anion.

Here, we introduce a general approach for modeling resonant photoelectron spectra of biological chromophores that allows us to account for and quantify the interplay between direct and resonant PD channels as a function of excitation wavelength. This also allows us to provide an important experiment-based reference for the adiabatic position of the lowest-lying excited shape (ES) resonance in HBDI^–^. Due to the one-electron nature of the decay process from an ES resonance, this state leads to most efficient autodetachment upon excitation above 3.49 eV (355 nm), right outside the S_0_ → S_1_ absorption band, and below 4.1 eV (300 nm), which corresponds to the opening of higher lying continua.^18,24^ We unravel the mechanism of resonant electron emission by accounting for changes in photoelectron distributions of HBDI^–^ upon excitation with photon energies within the range 355–315 nm, probing the entire S_0_ → ES resonance absorption band.

Beyond understanding the mechanism of electron emission, we show how the electron emission properties of the GFP chromophore can be tuned by coupling to a local environment. We address this by investigating electron emission from chemically modified deprotonated GFP chromophore anions in the gas phase. More specifically, we present new experimental photoelectron spectra at 328 and 346 nm of HBDI^–^ together with dimethoxy- (DMHBDI^–^) and difluoro-substituted (DFHBDI^–^) chromophores (Fig. 1) to investigate the influence of electron-donating and electron-withdrawing substituents on both the position of the detachment threshold and the electron emission mechanism. DMHBDI^–^ and DFHBDI^–^ are selected as biomimetics of the native GFP chromophore, as they have recently been shown to be fluorescent outside the native protein environment when bound to specific RNA sequences.^8^ These chemical modifications introduced to the GFP chromophore can be considered as perturbations for the intrinsic properties of HBDI and as such are likely to have implications for the function of these modified fluorophores. The combination of chemical tuning, photoelectron spectroscopy measurements and theory allows us to gain new insight into the light-induced electron emission properties of the GFP-related chromophores.

## Results and discussion

### Resonant electron emission from HBDI^–^

In the range 355–300 nm, right outside the S_0_ → S_1_ absorption band and below the opening of higher lying continua, HBDI^–^ has two valence-type ππ* states, 2^1^ππ* and 3^1^ππ*, located at 3.74 eV (331 nm) and 3.78 eV (328 nm), respectively (see Table 1). The first ^1^nπ* state is also located nearby, with the calculated vertical excitation energy (VEE) being equal to 3.43 eV (362 nm). The 3^1^ππ* (S_3_) state is a shape-type resonance, which corresponds to electron promotion from the highest occupied molecular orbital (HOMO) to the unoccupied orbital localized on the phenol ring (ESI Fig. S3†), and it is the first ES resonance that correlates with the ground state of the neutral radical D_0_ (ESI Fig. S4†). Autodetachment from this state has a one-electron nature.^18^ The other two states do not correlate with D_0_, and hence a two-electron process should be operative in autodetachment from these resonances, which have Feshbach character with respect to the D_0_ continuum. The 3^1^ππ* is thus most strongly coupled to the continuum and should result in most efficient autodetachment. Furthermore, the transition to this state has an appreciable oscillator strength.

**Table 1:** Calculated XMCQDPT2/(aug)-cc-pVTZ (Firefly (ref. 26)) and EPT/6-311++G(3df,3pd) (Gaussian 09 (ref. 27)) [in square brackets] vertical detachment and excitation energies (eV) of the chromophore anions^*a*^

		DMHBDI	HBDI	DFHBDI
S_1_	1^1^ππ*	2.46 (1.04)	2.62 (1.10)	2.53 (1.06)
D_0_	X(^2^A″)	2.69 [2.77]	2.74 [2.78]	2.99 [3.02]
S_1*n*_	1^1^nπ*	3.59 (0.00)	3.43 (0.00)	3.62 (0.00)
S_2_	2^1^ππ*	3.60 (0.00)	3.74 (0.02)	3.58 (0.00)
S_3_	3^1^ππ*	3.79 (0.00)	**3.78 (0.08)**	4.02 (0.02)
S_4_	4^1^ππ*	**4.08 (0.10)**	4.48 (0.01)	**4.20 (0.07)**
D_1*n*_	Ã(^2^A′)	4.05	3.97	4.47
D_1_	B(^2^A″)	4.35	4.40	4.66

^*a*^Highlighted are the ES resonances. Oscillator strengths are shown in parentheses. See also ESI† Tables S1–S3; XMCQDPT2 natural orbitals involved in electronic transitions are shown in ESI† Fig. S1–S4.

**Fig. 2:**
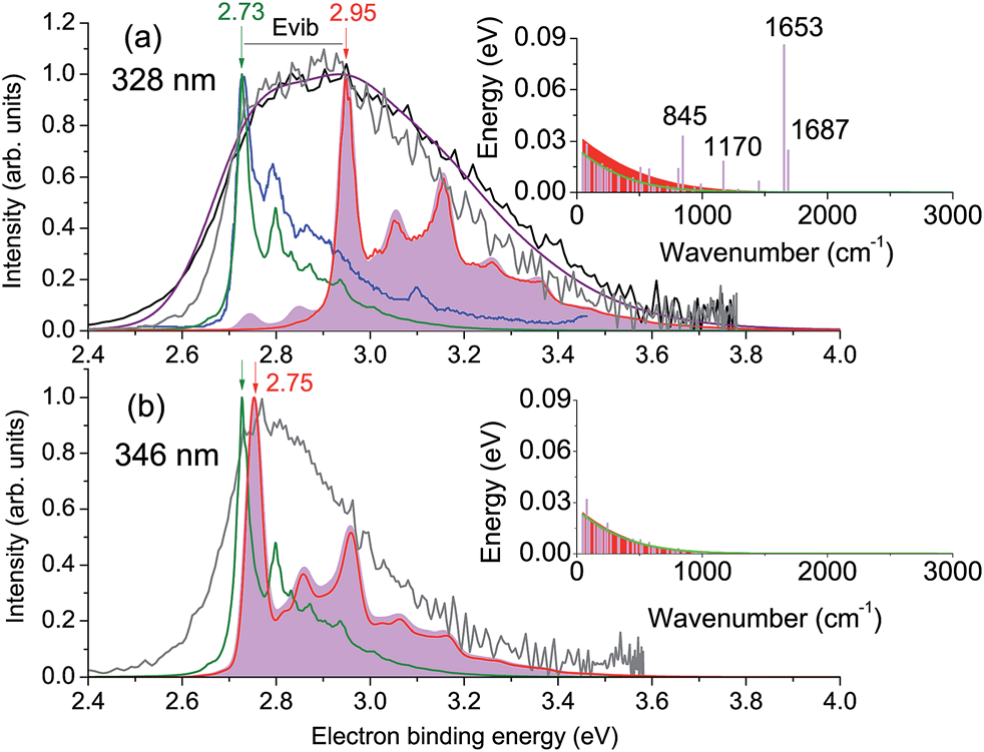
Photoelectron spectra of HBDI^–^ plotted as a function of electron binding energy, eBE = *hν* – eKE. Shown are the experimental spectra at (a) 328 nm and (b) 346 nm in gray (300 K, this work) and black (ref. 23), as well as the statistically calculated spectra of the direct S_0_ → D_0_ (green lines, 300 K) and resonant S_3_ → D_0_ (red lines, 400 K (a) and 300 K (b)) transitions. The purple line combines the direct and resonant channels in a 1.4 : 1.0 ratio, convoluted with a Gaussian function with a HWHM of 0.1 eV. The 0–0 onset of the S_0_ → D_0_ transition is placed at 2.73 eV to match the experimental value.^22^ Also shown is the 355 nm vibrationally resolved experimental spectrum (blue line in panel (a), 20 K (ref. 22)). The resonant parts calculated based on the initial Franck–Condon population of vibrational levels in the ES resonance are depicted as shaded violet. The insets show the average energies per each mode in S_0_ at 300 K before excitation (green line) and in S_3_ after excitation, with the initial (sticks) and equilibrated (∼400 K (a) and ∼300 K (b), shaded red) distributions.

The calculated vertical excitation energy to the 3^1^ππ* state is 3.78 eV, and the corresponding 328 nm experimental photoelectron spectrum of the deprotonated HBDI anion, plotted as a function of electron binding energy (eBE), is shown in Fig. 2a (Experimental details are described in ESI;† details of the photoelectron imaging apparatus can be found in ref. 20, 23, 28 and 29). The eBE distribution previously measured at a photon energy of 355 nm (3.49 eV) at ∼20 K (ref. 22) is also shown for comparison. The lowest overall cross-section in the action photoabsorption spectrum above the detachment threshold is observed at 355 nm,^18^ and, therefore, photoelectron spectra measured at this wavelength are not expected to be influenced by resonances.^19^ The low-temperature experiment eliminates spectral broadening due to hot transitions, both direct and resonant, thus revealing the most narrow, vibrationally resolved, eBE distribution that peaks at 2.73 eV (the 0–0 transition).^22^ The room-temperature experimental spectrum at 328 nm have a similar onset on the low eBE side, which is close to that of the low-temperature spectrum, and the low eBE side is thus assigned primarily to the direct S_0_ → D_0_ transition.

The D_0_ vertical detachment energies (VDEs) calculated using both extended multiconfiguration quasi-degenerate perturbation theory (XMCQPDT2)^30^ and electron propagator theory (EPT)^31,32^ methods are close to the experimental value of 2.73 eV (ref. 22) (Table 1). The calculated S_0_ → D_0_ spectral profile at 300 K (green curve, Fig. 2a), which is defined by the overlap of vibrational wave functions between ground anion and neutral states, agrees well with those previously calculated by us,^19^ as well as by other groups.^33^ It is also in a very good agreement with the experimental vibrationally-resolved spectrum taken at a photon energy of 355 nm (ref. 22) (blue curve in Fig. 2a, see also ESI Fig. S5–S7 and discussion on p. S20†).

**Fig. 3:**
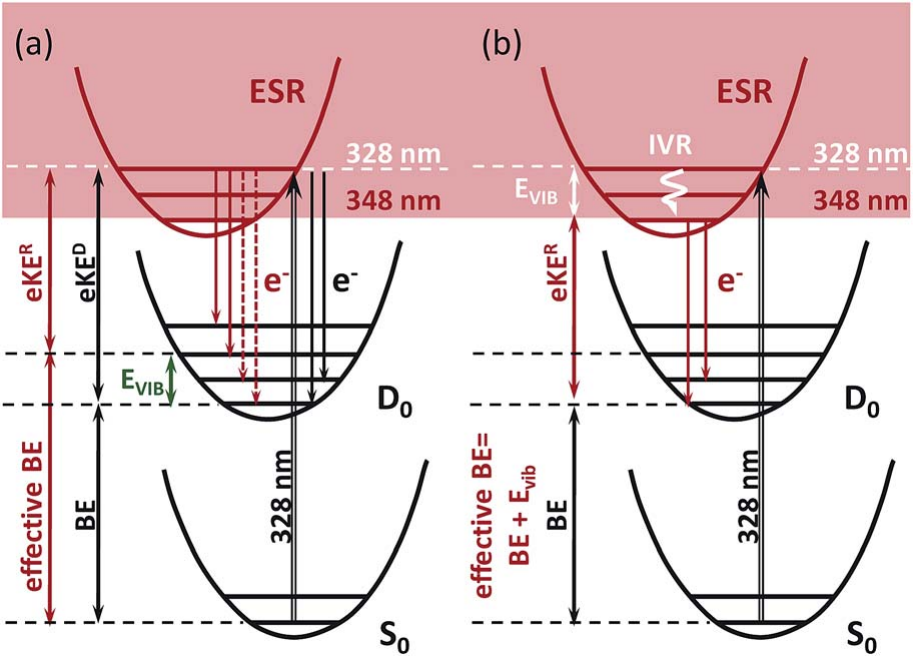
Direct S_0_ → D_0_ (black arrows) and resonant S_3_ (ESR) → D_0_ (red arrows) electron emission upon excitation at 328 nm. Shown are the true and effective electron binding energies (eBE) that correspond to direct photodetachment and autodetachment out of S_3_, respectively: (a) instantaneous and (b) delayed autodetachment. The difference in kinetic energy of the outgoing electrons emitted through the direct (eKE^D^) and resonant (eKE^R^) channels is equal to the energy stored in vibrations, *E*_vib_.

The 328 nm spectrum (Fig. 2a) is considerably broader on the high eBE side compared to those measured at 355 nm, thus confirming the presence of an indirect PD channel out of the ES resonance. The electron emission mechanism out of this state is illustrated in Fig. 3a (red arrows), as compared to direct PD (black arrows). Following S_0_ → S_3_ photoexcitation, the vibrational states in S_3_ are populated with a probability defined by the corresponding S_0_/S_3_ Franck–Condon overlap, and the photoelectron spectral shape of the resonant part is therefore defined as a product of the S_0_/S_3_ and S_3_/D_0_ Franck–Condon overlaps. The calculated resonant part at 328 nm based on the initial Franck–Condon population of vibrational levels in S_3_ is shown in Fig. 2a (shaded violet), and the inset shows the corresponding average energies per mode in S_3_ (sticks). The most active modes upon the S_0_ → S_3_ excitation are clearly identified as those that bear a substantial vibrational energy. When these modes are also Franck–Condon active upon autodetachment, they result in additional peaks, observed at 2.74 eV and 2.85 eV at 328 nm (1 → 0 transitions), below the most intense 0 → 0 (n → n) transition that peaks at an effective eBE of 2.95 eV (see also solid and dashed red arrows in Fig. 3a).

It is worth noting that the resonant shape calculated based on the initial S_0_/S_3_ Franck–Condon population of vibrational states is similar to that calculated based on the statistical population of vibrational levels in S_3_ (Fig. 2a, red line). The latter corresponds to a long-lived resonance, in which intramolecular vibrational energy redistribution (IVR) is complete and precedes autodetachment (see Fig. 3b). Similar spectral shapes of the resonant parts are due to the same S_3_/D_0_ origin shifts, and instantaneous autodetachment results in a photoelectron distribution that, although being broader due to a different Franck–Condon overlap between the excited vibrational levels in S_3_ and vibrational levels in D_0_, peaks at the same effective eBE, as in the case of delayed autodetachment (Fig. 3a and b). This is true when not so high vibrational levels of the Franck–Condon active modes are populated following the S_0_ → S_3_ excitation (see ESI p. S23 and Fig. S8†).

As a result, regardless of a particular lifetime of the ES resonance and a timescale of IVR, autodetachment out of this state is characterized by a shifted onset in the photoelectron distribution. This shift corresponds to the excess of vibrational energy gained upon resonant photoexcitation that is subsequently stored in neutral radicals upon the S_3_ → D_0_ transition. The resonant photoelectron distribution is shifted as a whole to lower kinetic energies of the outgoing electrons (eKE^R^) with respect to those of the direct channel (eKE^D^). Therefore, the higher effective eBE at 2.95 eV corresponds to a sum of a true eBE at 2.73 eV and a fraction of a photon energy stored in vibrations (*E*_vib_, see Fig. 2a). Furthermore, due to the shape character of the ES resonance, the interaction of this state with the electron continuum is large, and the direct transition to the continuum can thus borrow intensity from the optically bright excitation in the anion.^34^ Both direct and indirect PD channels are operative at the excitation wavelengths resonant with the S_3_ state.

A major source of the large width observed in the experimental photoelectron spectrum of HBDI^–^ at 328 nm with respect to that registered at 355 nm is, therefore, due to a superposition of the S_0_/D_0_ and S_3_/D_0_ spectra with the shifted onsets. Since the direct and resonant parts both peak at the 0 → 0 transition (see ESI Fig. S6 and S9†), the shift is defined as a difference between the energies of a photon and a zero vibrational level of the ES resonance with respect to S_0_. Fig. 2a shows the statistically calculated direct and resonant parts, shifted by 0.22 eV at 328 nm (3.78 eV), which corresponds to the calculated S_0_ → S_3_ vertical excitation energy (Table 1). This shift is consistent with the calculated relaxation energy of 0.2 eV in S_3_, and the combined spectrum with almost equal weights for the two parts reproduces the experimental shape. From this, the adiabatic location of the ES resonance with respect to S_0_ is obtained and is found to be 3.56 eV (348 nm).

The experimental photoelectron spectrum at an excitation energy of 346 nm, which is very close to the obtained adiabatic S_0_ → S_3_ transition (348 nm), is consistent with the calculated direct and resonant parts (Fig. 2b). In this case, the onsets of the two parts nearly coincide in energy and the experimental spectral width is defined by the broader resonant shape. This supports our theoretical model of the resonant photoelectron spectra of HBDI^–^.

In HBDI^–^, resonant PD has previously been found to occur within 55 fs,^24^ which is consistent with the shape resonance nature of the S_3_ state.^35,36^ Although the vibrational relaxation dynamics in the ES resonance is out of the scope of the present study, we note that the initial vibrational relaxation may compete with autodetachment from a given vibrational level in S_3_.‡
‡The relaxation characteristics of a single 0 → 1 excited mode coupled to a bath of remaining modes in S_3_ can be estimated using the ‘golden rule’.^37^ In HBDI^–^, the calculated density of the Fermi resonances is ∼7 × 10^4^ per cm^–1^ for a high-frequency stretching mode of 1653 cm^–1^, which is active in photoexcitation as well as autodetachment from the ES resonance. Assuming small effective interaction strengths of 0.09–0.04 cm^–1^, the initial relaxation time is on the order of 10–50 fs for transferring energy from the initially excited state to vibrational states at the same energy. The nuclear dynamics in S_3_ may thus represent an additional source for broadening of each transition within a vibrational manifold of HBDI^–^, in addition to a nuclear lifetime broadening (*i.e.* vibrational decoherence) in the final D_0_ state. In the following, we use statistically calculated direct and resonant parts and convolute both parts with a Gaussian function with a half width at half-maximum (HWHM) of 0.1 eV. By doing this, we take into account many low-intensity transitions from higher vibrational states in S_3_ populated upon the S_0_ → S_3_ excitation as well as a nuclear lifetime broadening in the resonance state. The same Gaussian function is subsequently used to model spectral shapes at different excitation wavelengths within the entire S_0_ → S_3_ absorption band, indicating a similar line broadening mechanism.

### Scanning through the shape resonance of HBDI^–^

High-energy photoexcitation within the S_0_ → S_3_ absorption band results in simultaneous excitation of a number of active Franck–Condon modes, and a substantial fraction of the photon energy can thus be stored in many vibrational degrees of freedom of the chromophore. This results in a lack of the normal photoelectric effect, *i.e.* a shift in photoelectron distributions to higher eKE with increase of photon energy, for the resonant part of the photoelectron spectrum, while the direct part follows this law, as expected.

**Fig. 4:**
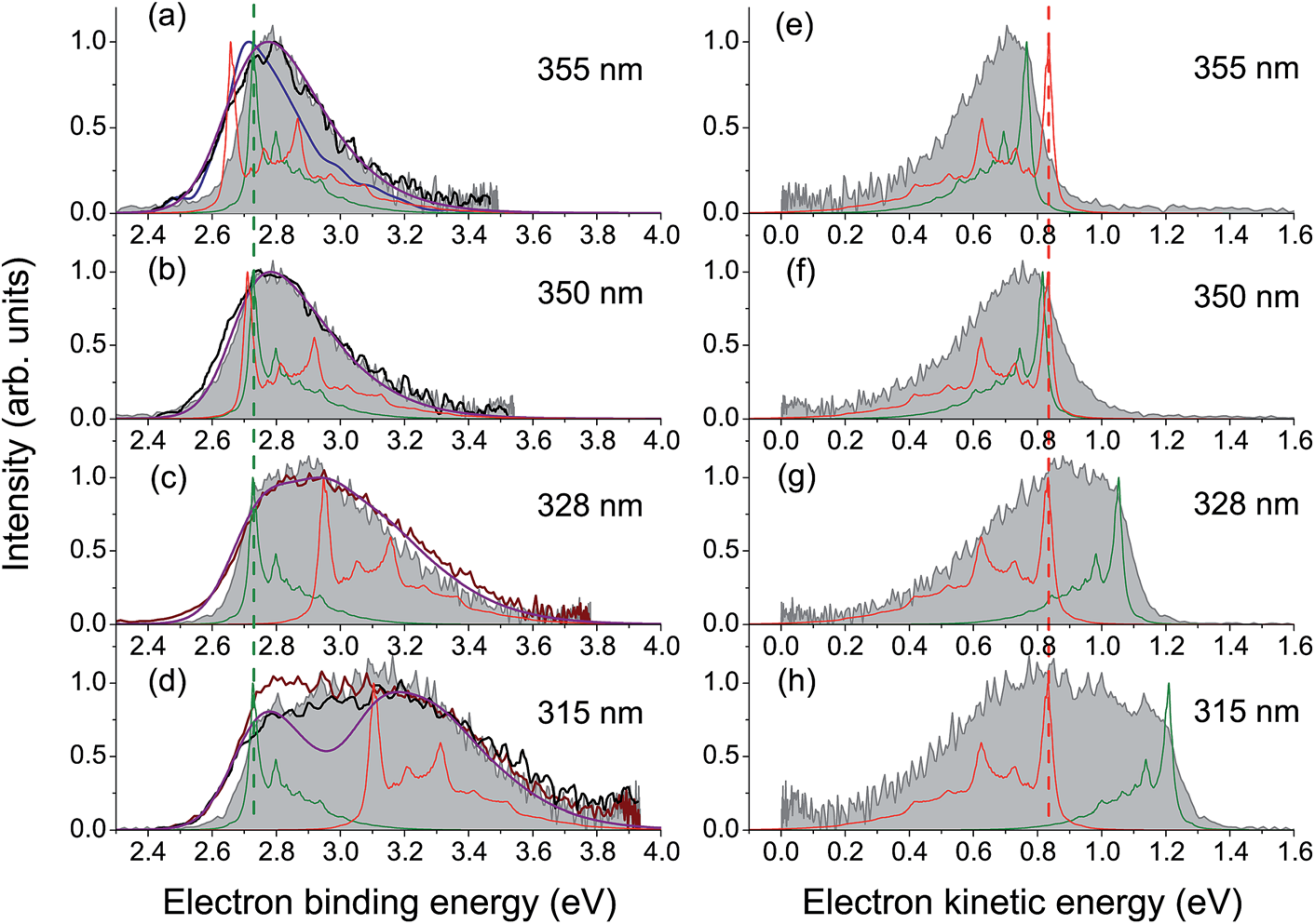
Experimental and theoretical photoelectron spectra of HBDI^–^ obtained at various wavelengths within the S_0_ → S_3_ absorption band. The spectra in panels (a)–(d) are plotted as a function of eBE. The experimental spectra presented in shaded gray are from the present work. The photoelectron distributions shown in brown are from ref. 23 (328 and 315 nm), in black – from ref. 24 (355, 350 and 315 nm), and in blue – from ref. 19 (355 nm). Depicted are also the statistically calculated direct (green, 300 K) and resonant (red, 300 K at 355 and 350 nm and 400 K at 328 and 315 nm) parts, as well as the combined and convoluted theoretical spectra (purple). The direct/resonant PD ratios are in the range of 1.1–1.4. In panels (e)–(h), the same spectra are plotted as a function of eKE. Note that the direct part defines a true eBE (a–d, green dashed line), while the resonant part peaks at a constant eKE (e–h, red dashed line). For variations in the experimental and theoretical spectra, see ESI pp. S27–S28 and Fig. S10.†

In Fig. 4, experimental and theoretical photoelectron spectra of HBDI^–^ at various excitation wavelengths within the S_0_ → S_3_ absorption band are shown. In panels (a)–(d), all photoelectron spectra are plotted as a function of eBE, and both the calculated individual parts and the resulting shapes are presented, where the latter are remarkably consistent with the experimental spectral profiles. The adiabatic position of the ES resonance and a photon energy define the shifts between the origins of the two individual spectral components. The direct part, shown in green, always peaks at a true eBE of 2.73 eV, while the resonant part, shown in red, peaks at effective BEs defined by the correspondingly larger fractions of increasing photoexcitation energy stored in vibrations. The shift between the two parts, which becomes larger with increasing excitation energy, results in the progressively broader eBE distributions. The same individual spectral profiles plotted as a function of eKE are presented in panels (e)–(h).

Since the progressively larger widths of the resonant photoelectron spectra measured within the S_0_ → S_3_ absorption band can be fully accounted for taking into account direct PD and autodetachment out of the ES resonance only, we conclude that relaxation through internal conversion to lower-lying excited states does not play a significant role in PD from HBDI^–^ following its resonant excitation to the S_3_ state, contrary to what has been suggested recently.^24^ Indeed, the ultrashort excited-state lifetime of 55 fs (ref. 24) is more consistent with the shape character of an autodetaching resonance, than with the Feshbach character of the lower-lying S_2_ state. The absence of the characteristic peak of thermionic emission from the anionic hot ground state,^24^ as well as a lack of any delayed signal in time-domain action-absorption spectroscopy experiments,^18^ indicates that the HBDI^–^ excited-state lifetime is limited by electron emission, rather than by internal conversion back to the ground state. The abrupt change in the photoelectron distribution of HBDI^–^ right above 4.1 eV (∼300 nm)^24^ can be explained by the opening of the D_1*n*_ electronic continuum (VDE of 3.97 eV, see Table 1), which becomes visible at 315 nm (3.94 eV) as a zero-eKE peak (Fig. 4h), and hence a new direct one-electron decay channel becomes available. At the same time, resonant PD through the ES resonance plays a less significant role above 4.1 eV as the S_0_ → S_3_ absorption gets weaker (the calculated full width at half maximum of the absorption band is 0.4 eV).

### Tuning the electron emission properties of HBDI^–^

By introducing chemical modifications to the structure of the chromophore, the electron detachment thresholds and the position of the ES resonance can be changed systematically. Here, we consider difluoro- and dimethoxy-substituents of HBDI^–^ at the *ortho* positions in the phenol ring (Fig. 1); the fluoro substituents have an overall σ electron-withdrawing effect, whereas the methoxy substituents have overall π electron-donating character. By introducing these substitutions to HBDI^–^, we might therefore expect to reveal opposite shifts in energy of the D_0_ threshold, since the ability of the chromophore anion to support an electron depends on stabilizing or destabilizing the entire electron density. In contrast, nπ* and ππ* excitation energies should primarily be sensitive to individual σ- or π-effects, depending on the character and symmetry of orbitals involved in the transitions (A′ or A″ with respect to the plane of the chromophore).

**Fig. 5:**
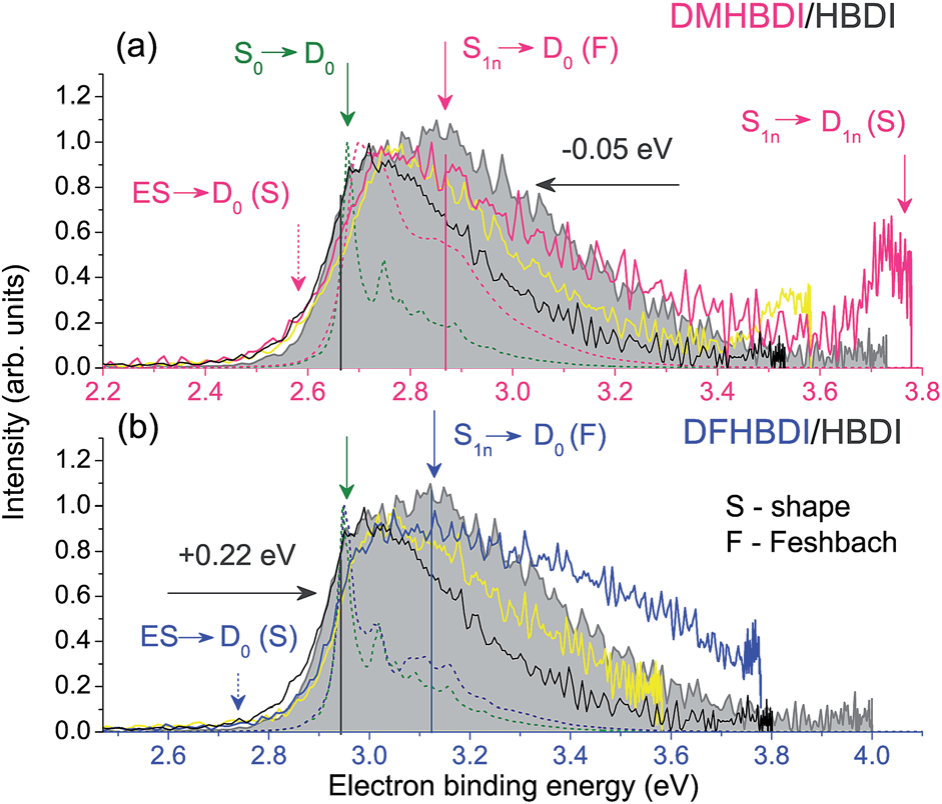
Photoelectron spectra of the substituted chromophore anions, DMHBDI^–^ (a) and DFHBDI^–^ (b). Shown are the experimental eBE distributions at 328 nm (pink and blue lines) and 346 nm (yellow lines) superimposed with those of HBDI^–^ at 328 nm (shaded gray) and 346 nm (black line). The HBDI^–^ spectra are shifted by –0.05 eV and +0.22 eV to match the low eBE side of the spectral shapes of DMHBDI^–^ and DFHBDI^–^, respectively. The calculated spectral profiles at 300 K for the direct S_0_ → D_0_ transition are depicted as dashed lines in pink (DMHBDI^–^), blue (DFHBDI^–^), and green (HBDI^–^). Vertical positions of the S_1*n*_ states are shown as vertical lines, calculated as shifts from the true eBEs. The dashed arrows indicate the estimated adiabatic positions of the ES resonances of the substituted chromophores, which are unlikely to be populated at these excitation wavelengths, in contrast to HBDI^–^.

The 328 nm photoelectron spectra of the substituted chromophore anions are shown in Fig. 5. Adding π electron-donating methoxy substituents to the phenoxide moiety of HBDI^–^ results in a red-shift (∼0.05 eV) of the photoelectron spectrum. Conversely, adding σ electron-withdrawing substituents results in a blue-shift (∼0.22 eV). At the same time, all three chromophores have photoelectron distributions with similar shapes on the low eBE side. When correspondingly shifted, the low-energy parts of the DMHBDI^–^ and DFHBDI^–^ distributions practically coincide with that of HBDI^–^. Since in the latter case, the onset is defined by the direct S_0_ → D_0_ transition (see Fig. 2), an impact of resonance states at the eBE onsets of DMHBDI^–^ and DFHBDI^–^ is expected to be small. From this, we are able to determine that the adiabatic detachment thresholds of DMHBDI^–^ and DFHBDI^–^ are located at 2.68 and 2.95 eV, respectively (see also ESI Fig. S11†).

The calculated VDEs (see Table 1) are consistent with the experimental photoelectron spectra and can be rationalised by considering the resonance structures of the anions and their corresponding neutral radicals (see ESI Fig. S12 and S13†). For the anion, increased electron density at the *ortho* positions on the phenoxide group causes the electron-withdrawing fluoro substituents to lower the energy of the anion and the electron-donating methoxy groups to raise the energy of the anion. For the radical, both electron-withdrawing and electron-donating substituents will lower the energy by resonance stabilization. However, one of the resonance structures of the methoxy substituted radical is a stable captodative radical, suggesting that the methoxy-substituted radical will be lowered in energy more than the fluoro-substituted radical. This leads to the S_0_–D_0_ energy difference increasing in the order DMHBDI^–^ < HBDI^–^ < DFHBDI^–^, which is consistent with the trend in VDE.

As well as providing information about the photon energy required to induce electron emission, the photoelectron spectra provide information about the wavelength-dependent electron emission mechanism. As expected, the eBE distributions at 328 nm, which is ∼1 eV above their detachment thresholds, are broad, indicating the involvement of resonance states in PD from both DMHBDI^–^ and DFHBDI^–^. The DMHBDI^–^ and DFHBDI^–^ chromophore anions have higher VEEs to the ES resonance, compared to HBDI^–^ (see Table 1). The large shift of 0.3–0.4 eV is due to significant destabilization of the highly localized unoccupied 
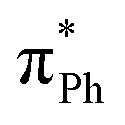
 orbital (ESI Fig. S14†) involved in the π_HOMO_ → 
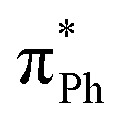
 transition, where the electron density moves from the allyl bridge to the phenol ring. In contrast, a counterpart to this excitation, the 
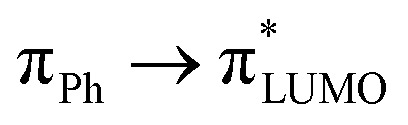
 transition, drops in energy, since the occupied π_Ph_ orbital is now destabilized. This causes a flip of the 3^1^ππ* and 4^1^ππ* states in the substituted chromophore anions.

**Fig. 6:**
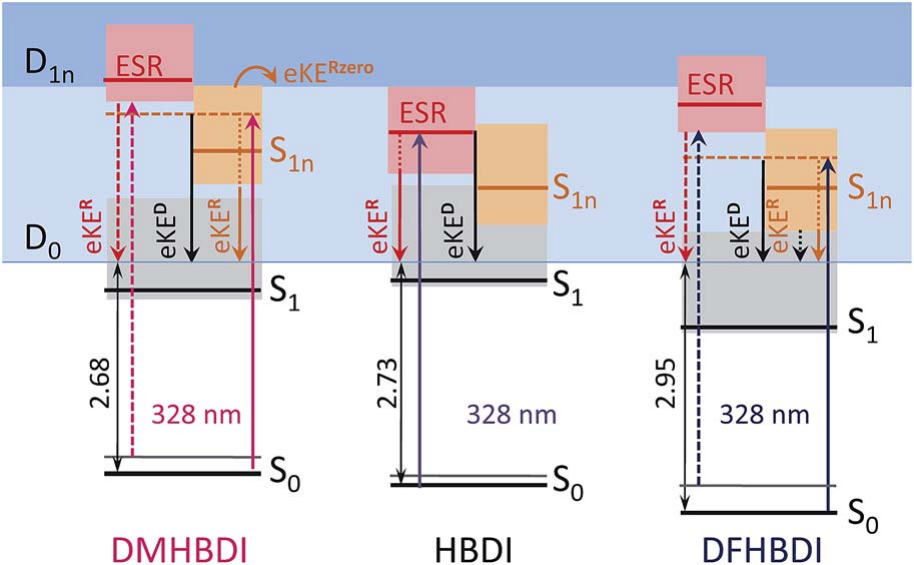
Energy-level diagram of the excited states in the deprotonated chromophores with respect to the electron continuum. The experimental detachment thresholds and the calculated VEEs and higher-lying VDEs are used (see Table 1). The shaded areas stand for vibrational manifolds. For clarity, the D_1*n*_ continuum is shown at a constant level for all anions at 1.4 eV above D_0_.

Shifted to higher energies by 0.3–0.4 eV, it is unlikely that the ES resonances located vertically at 4.08 (DMHBDI^–^) and 4.20 eV (DFHBDI^–^) are significantly populated at 328 nm (3.78 eV). The impact of the ES resonance may only appear at the low eBE side of the 328 nm photoelectron spectrum of DMHBDI^–^ enabled through hot transitions upon resonant photoexcitation, while the presence of this state is even less likely for DFHBDI^–^, since more energy has to be borrowed from ground-state vibrations to reach it (Fig. 5 and 6).

On the high eBE side of the 328 nm photoelectron spectra of the substituted chromophore anions, we expect to see fingerprints of lower-lying Feshbach resonances, in particular, the 1^1^nπ* states shifted in energy from 3.43 eV (HBDI^–^) to 3.59 (DMHBDI^–^) and 3.62 eV (DFHBDI^–^) (Fig. 6). In contrast to π orbitals, totally symmetric n orbitals experience a σ electron withdrawing effect from the substituents and become more stabilized. The S_0_–S_1*n*_ excitation energy therefore increases in the order HBDI^–^ < DMHBDI^–^ < DFHBDI^–^. Transitions to the 1^1^nπ* states are known to gain their intensity through vibronic couplings.^35^

The 328 nm spectrum of DMHBDI^–^ also exhibits a peak at 3.78 eV (Fig. 5), which corresponds to a kinetic energy of the ejected electrons being close to zero, thus indicating the opening of a higher-lying electron continuum. The D_1*n*_ vertical detachment threshold lies just above 4.0 eV (see Table 1) and can be reached through autodetachment out of the 1^1^nπ* state that has a shape character with respect to this higher-lying continuum. Being a Feshbach resonance with respect to the open D_0_ continuum, this state is expected to live long enough to enable decay to D_1*n*_, thus supporting our assignment. In DFHBDI^–^, this continuum lies ∼0.5 eV higher in energy, and hence it cannot be reached even through vibrationally assisted autodetachment out of the 1^1^nπ* state upon excitation at 3.78 eV, which is consistent with the experimental data. In the absence of this vibrationally assisted shape-type autodetachment channel, nuclear relaxation and internal conversion to lower-lying excited-states may be operative in DFHBDI^–^, as evidenced by a significantly higher amount of the ejected electrons with a very broad distribution at the high eBE side of the 328 nm photoelectron spectrum (Fig. 5b).

Finally, according to our general approach we might expect to get narrower photoelectron distributions of the substituted chromophores when decreasing excitation energy from 3.78 eV (328 nm) to 3.58 eV (346 nm), so that the excitation energy is very close to the calculated vertical excitation energies to the low-lying 1^1^nπ* states in DMHBDI^–^ and DFHBDI^–^ (see Table 1). This is indeed the case and the experimental 346 nm photoelectron distributions are shown in Fig. 5 (yellow lines). The high eBE sides of these spectra are narrower due to smaller shifts between the onsets of the direct and 1^1^nπ* resonant parts. Despite rather similar shapes of the 346 nm photoelectron distributions of all chromophores, the photoelectron spectrum of HBDI^–^ is dominated by the presence of the ES resonance, with the excitation energy being close to the adiabatic transition to this state (Fig. 2b).

Based on our theoretical account of the experimental photoelectron spectra and the calculated large shifts of the ES resonances to higher energies (0.3–0.4 eV), we conclude that the mechanism of resonant PD at 328 nm is different in the deprotonated biomimetic GFP chromophores compared to that found in the native GFP chromophore anion, where this excitation wavelength coincides with the vertical excitation energy to the ES resonance. The difluoro- and dimethoxy-substitutions are remarkable, since they have opposite affects on the electron detachment threshold in HBDI^–^, whereas the positions of the resonance states undergo shifts in the same direction; enabling greater flexibility in tuning the intrinsic properties of the GFP chromophore anion acting as a light-induced electron donor, with respect to both the photon energy required to initiate electron emission and the mechanism of electron detachment. Beyond the present case, our work shows that the detailed knowledge of the pattern and positions of the excited states are essential in revealing the light-induced electron emission properties of biological chromophores, since their photoelectron spectra are dominated by resonances.

### Implications to biological GFP and RNA environments

**Fig. 7:**
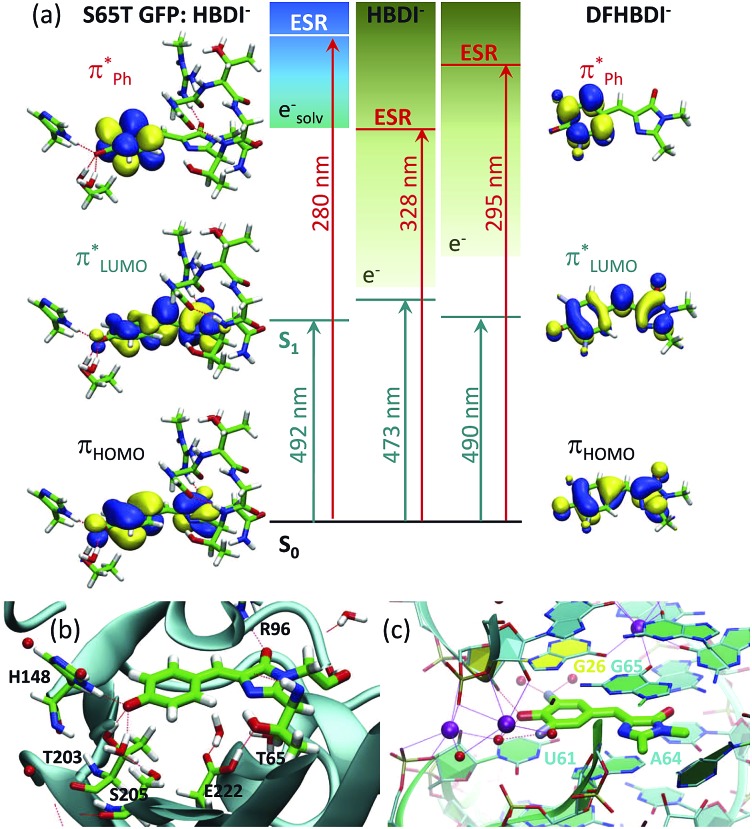
Tuning the intrinsic photophysical properties of the GFP chromophore anion through coupling to environment. (a) Shifts in vertical excitation and detachment energies induced by the S65T GFP protein and by the fluoro-substituents. The XMCQDPT2 natural orbitals involved in the S_0_ → S_1_ (
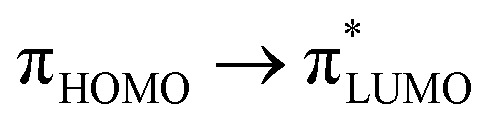
) and S_0_ → ES resonance (ESR, 
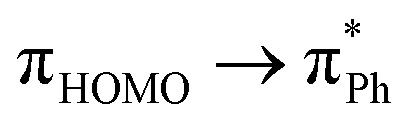
) excitations are shown. (b and c) Biological environments of HBDI^–^ in S65T GFP (b) and of DFHBDI^–^ in the RNA–fluorophore complex (c). The QM/MM optimized structure of S65T GFP (PDB 1EMA (ref. 38)) and the crystal structure of the spinach–DFHBDI^–^ complex (PDB 4TS0 (ref. 39)) are presented. Color code stands for the C, N, O, H, F atoms depicted in green, blue, red, white, and light blue, respectively. Red and purple spheres represent water and K^+^, respectively. H-Bonds are depicted as red lines. Cation coordination is shown in purple lines. The protein calculations are from ref. 18.

A pattern of the excited states and their nature with respect to electron removal in the GFP chromophore anion is important for understanding the mechanism of resonant electron transfer from the deprotonated chromophore inside the protein and, ultimately, for enhancing or preventing its photooxidation. In this respect, the ES resonance, which is found to be strongly coupled to the open electron continuum in the gas phase, is particularly important. Due to the one-electron nature with respect to electron removal, resonant electron transfer out of this state in GFP, among all other excited states S_*n*_ (*n* > 1) within the same UV energy region, should also be most efficient. The calculated vertical excitation energy to the ES resonance is shifted from 3.8 eV (328 nm) in the isolated anion to 4.4 eV (280 nm) in the S65T GFP protein, and this state is, therefore, resonant with a quasi-continuum of a hydrated electron^18^ (see Fig. 7).

Remarkably, the ES resonance of the deprotonated GFP chromophore anion inside the protein lies within the UV energy range, excitation to which results in the oxidative decarboxylation of GFP.^13^ This indicates that the primary step in the photoconversion mechanism might be a light-driven formation of the neutral radical chromophore, where the excited GFP chromophore anion first photooxidizes through this state upon one-photon excitation in the UV or two-photon absorption in the visible range. The neutral radical chromophore can be a common oxidant state for all GFP-like proteins, where the light-driven oxidative decarboxylation has been observed. Upon irradiation with visible light, within the A-band of wild-type GFP (390 nm (ref. 12) or 404 nm (ref. 13)), the neutral chromophore may first undergo ultrafast excited-state proton transfer, which is well known to be operative in GFP,^40^ followed by sequential absorption of a second photon from the first excited state of the anion, within at least a nanosecond duration of the laser pulse with a sufficient photon flux. Naturally, this mechanism needs to be studied experimentally, using, for example, ultrafast transient absorption spectroscopy to identify short-lived radical intermediates and a solvated electron. Also, the photoconverted GFP, which lacks a primary redox partner for the transiently formed neutral radical chromophore, a decarboxylated residue E222, can be studied upon UV irradiation to analyze the possibility of photooxidation of the anionic GFP chromophore that results in presumably longer-lived radical species. Upon excitation with a 4 ns 355 nm laser pulse, formation of the solvated electron has indeed been observed in the photocycle of mKalama1, containing the GFP chromophore in the neutral form, enabled through two consecutive resonant absorption events.^41^ This protein contains a key V224R substitution in the binding pocket of the chromophore that blocks excited-state protein transfer and immediate redox reactions with E222.

The fluoro- and methoxy-substituents introduced to the GFP chromophore act as perturbations for the intrinsic photophysical properties of HBDI^–^, and their effect on the electronic structure of HBDI^–^ can thus be considered as a local environmental effect. By analyzing changes in the pattern of the excited states of the GFP chromophore anion and its chemically modified analogues, important information can be gained for predicting the light-induced redox properties of DFHBDI^–^ and DMHBDI^–^. As discussed above, we consider the ES resonance to be an electron gateway state for resonant electron transfer from the deprotonated chromophores. This state is significantly shifted to higher energy in the biomimetic chromophores (see Fig. 7), and higher energy photons will subsequently be required to initiate resonant electron transfer out of this state to the solvent, when the modified chromophores are immersed into the media. This suggests that the biomimetic GFP chromophores are more protected against UV photooxidation in media and, in particular, in their biological RNA environment. The light-induced redox properties of recently discovered RNA–fluorophore complexes, such as spinach–DFHBDI^–^,^7^ remain to be resolved experimentally.

Light-induced electron transfer is at the heart of many redox processes in biology. By uncovering the role of electronically excited states in the light-induced electron emission properties of the GFP chromophore and its biomimetics, this combined theoretical and experimental study provides a general understanding of electron emission from biological chromophores over a wide range of excitation energies. This has significant implications to biological environments, as we demonstrate that the positions and the nature of the excited states in the isolated chromophores are essential as references for those perturbed by their environments. And as such, we believe that our results provide an important step toward the experimental study of the complex electron transfer mechanisms in GFP and RNA–fluorophore complexes.

## Conclusions

This combined experimental and theoretical study has allowed us to disentangle the photoinduced electron emission dynamics of the GFP chromophore anion above the detachment threshold following 355–315 nm excitation, where many excited states and competing non-adiabatic decay channels such as internal conversion may contribute. We show that the ultrafast electron dynamics is primarily governed by a single, optically bright, excited shape resonance, which is strongly coupled to the open electron continuum. Importantly, we provide a well-defined reference for the position of the electron gateway state in the GFP chromophore anion and show that the electron emission mechanism through this state is inherently dual – both the direct and resonant transitions to the electron continuum plays here a significant role. When scanning through the shape resonance with increasing excitation energy, the photoelectron distribution therefore undergoes a significant broadening. This is well captured and accounted for by our proposed theoretical approach.

Based on the wavelength-dependent PD mechanism, we show that the intrinsic electron emission properties of the deprotonated GFP chromophore can be tuned through coupling to a local environment, here introduced as chemical modifications. The difluoro- and dimethoxy-substituents affect both the photon energy required to initiate electron emission and the mechanism of electron detachment. A resonant mechanism has been found in PD from all chromophores at 328 nm that coincides with the vertical excitation energy to the electron gateway state in the native GFP chromophore; however, the nature of the resonances is different in the modified chromophores. Based on a considerable blue shift of the electron gateway state, the biomimetic chromophores are suggested to be more stable to photooxidation in media, in particular in their biological environments, since higher energy photons are required to initiate resonant electron transfer.
