# Supplementary material for: Mechanism of resonant electron emission from the deprotonated GFP chromophore and its biomimetics
Source: Chem Sci. 2017 Feb 6;8(4):3154–63. doi: 10.1039/c6sc05529j (PMC5413970; doi:10.1039/c6sc05529j)
Supplement: Supplementary file 1 [file SC-008-C6SC05529J-s001.pdf]

Electronic Supplementary Material (ESI) for Chemical Science.
This journal is © The Royal Society of Chemistry 2017

ELECTRONIC SUPPLEMENTARY INFORMATION

Mechanism of resonant electron emission from the deprotonated GFP chromophore and its biomimetics

Anastasia V. Bochenkova,[*,†] Ciarán R.S. Mooney,[‡] Michael A. Parkes,[‡] Joanne L. Woodhouse,[‡] Lijuan Zhang,[‡] Ross Lewin,[‡] John M. Ward,[¶] Helen C. Hailes,[‡] Lars H. Andersen,[§] and Helen H. Fielding[*,‡]

Department of Chemistry, M.V. Lomonosov Moscow State University, 119991 Moscow, Russia, Department of Chemistry, University College London, 20 Gordon Street, London WC1H 0AJ, U.K., Department of Biochemical Engineering, UCL, Bernard Katz Building, Gordon Street, London, WC1E 0AH, UK., and Department of Physics and Astronomy, Aarhus University, DK-8000 Aarhus C, Denmark

E-mail: bochenkova@phys.chem.msu.ru; h.h.fielding@ucl.ac.uk

[*]To whom correspondence should be addressed
[†]Department of Chemistry, M.V. Lomonosov Moscow State University, 119991 Moscow, Russia
[‡]Department of Chemistry, University College London, 20 Gordon Street, London WC1H 0AJ, U.K.
[¶]Department of Biochemical Engineering, UCL, Bernard Katz Building, Gordon Street, London, WC1E 0AH, UK.
[§]Department of Physics and Astronomy, Aarhus University, DK-8000 Aarhus C, Denmark

Contents

1 **Experimental methods** **S3**

2 **Computational details** **S3**

3 **Calculated vertical excitation energies in the anions** **S9**

4 **Calculated higher-lying vertical detachment thresholds** **S11**

5 **Character of the electronic transitions** **S12**

6 **$S_0 \rightarrow D_0$ photoelectron spectral shape in PD from $HBDI^-$** **S18**

7 **Autodetachment out of S_1 above the S_1/D_0 crossing** **S20**

8 **$ESR \rightarrow D_0$ photoelectron spectral shape in PD from $HBDI^-$** **S23**

9 **Variations in experimental and theoretical spectra** **S27**

10 **Active modes in direct PD from $DMHBDI^-$ and $DFHBDI^-$** **S29**

11 **Impact of the substituents** **S30**

12 **XYZ coordinates of the optimized structures in Å** **S33**

1 Experimental methods

The GFP chromophore and its biomimetics are synthesised using established methods,[1] *via* synthesis of the azalactone and subsequent aminolysis with methylamine. They are purified to remove minor contaminants by recrystallisation from ethanol. Photoelectron spectra of the deprotonated chromophore anions are recorded using anion photoelectron velocity-map imaging. The deprotonated chromophore anions are generated by electrospray ionisation of HBDI, DMHBDI, or DFHBDI, dissolved in methanol with a few drops of NH_3. The deprotonated chromophore anions are mass-selected by a quadrupole, trapped in a hexapole trap and focused into the centre of a velocity-map imaging apparatus. Subsequently, these anions are irradiated with nanosecond pulses of 328 nm (3.78 eV) light generated by frequency-doubling the output of a Nd:YAG pumped dye laser system. Photoelectrons are focussed onto a position sensitive detector. Background counts from anions and stray laser light are removed by subtracting anion-only and laser-only images from images recorded following interaction between the laser light and anions. These background-subtracted images are then processed using the pBasex method.[2] The spectrometer is calibrated by recording the photoelectron spectrum of I^-. The resolution of the spectrometer is $\sim 5\%$, which corresponds to a full-width at half-maximum (FWHM) of 0.05 eV at 1 eV eKE. Additional details of the anion photoelectron imaging apparatus have been published elsewhere.[3–6]

2 Computational details

The MP2/(aug)-cc-pVTZ level of theory, with the cc-pVTZ basis set augmented by a diffuse *spdf* shell on all oxygen atoms, is employed for optimizing geometry parameters of the deprotonated chromophore anions in the ground electronic state. Vibrational analysis is used to verify that the stationary points found are true minima on the corresponding potential energy surfaces. Equilibrium geometries of the neutral chromophore radicals, anionic excited-state gradients, vertical excitation and detachment energies are calculated using the extended multi-configuration quasi-

degenerate perturbation theory, XMCQDPT2,[7] within the same valence-type basis set. The reference wave functions of the ground and target states are constructed within the complete active space self-consistent field method using a state-averaging procedure, SA-CASSCF. Within a large basis set used in the present work, anionic vertical excitation energies and excited-state gradient calculations are performed using a DFT/PBE0-based (rather than a closed-shell Hartree-Fock-like) one-electron Fock-type matrix, when obtaining energies of all MCSCF semi-canonical orbitals used in a perturbation theory series. This is done in order to improve a description of open-shell states in the anions. The Firefly package[8] has been used for all electronic structure calculations.

Vertical $\pi\pi^*$ excitation energies (VEE) of $HBDI^-$ are calculated at the XMCQDPT2/SA(7)-CASSCF(16,14)$^\pi$/(aug)-cc-pVTZ level of theory within the active space, which comprises all valence π-type orbitals. The XMCQDPT2 effective Hamiltonians are constructed in the frame of the reference spaces spanned by 7 SA(7)-CASSCF or 10 CASCI wavefunctions. Vertical $\pi\pi^*$ transition energies in the substituted chromophore anions, $DMHBDI^-$ and $DFHBDI^-$, are calculated within the same active space as in $HBDI^-$, as well as within the larger active space that also includes a linear combination of π-type lone pairs at the substituents, XMCQDPT2/SA(7)-CASSCF(18,15)$^\pi$. Vertical nπ^* excitation energies in all chromophore anions are obtained within the mixed active spaces modified in order to include the n orbital of the phenoxide oxygen, whereas one occupied π-orbital with the largest occupation number is kept as doubly occupied during the CASSCF procedure. The excluded π orbital refers to a lone pair of electrons predominantly localized at the nitrogen atom of the amino group of the heterocyclic ring. Only the ground and low-lying $\pi\pi^*$ and nπ^* states are included in the state averaging procedure at the XMCQDPT2[7]/SA(3)-CASSCF(16,14)$^{\pi/n}$ level of theory. The orbitals included in the CASSCF active spaces are illustrated in Figure S1. It is worth noting, that we anticipate effects of the continuum, which are not included here, on line shapes and positions of, in particular, shape resonances of the isolated anions in the UV. The line widths due to electron lifetime broadening are expected to be up to 200 cm^{-1},[9] which do not alter the pattern of bound and quasi-bound excited states.

Vertical detachment energies (VDEs) of the anions are calculated at the XMCQDPT2[7]/

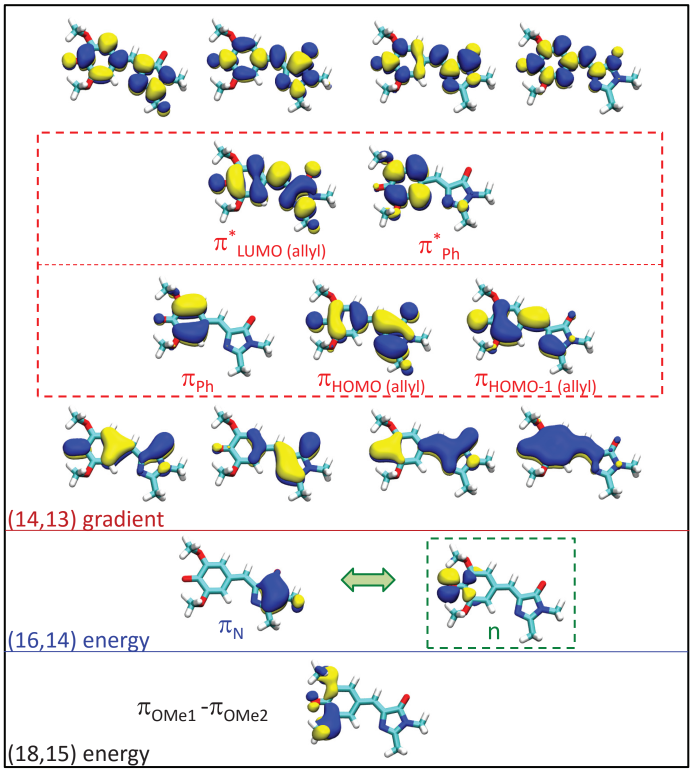

Figure S1: Orbitals of the active spaces used in the present work. Exemplified are the XMC-QDPT2/CASSCF(18,15) π-type and XMCQDPT2/CASSCF(16,14) n-type zeroth-order natural orbitals of the ground electronic state in DMHBDI^{-}. Pure π-type active spaces include either 18 electrons and 15 orbitals or 16 electrons and 14 orbitals. The $n \rightarrow \pi^{*}$ transitions are calculated within (16,14) active spaces, where one π-type orbital is replaced with the n orbital. The gradient in the anionic ESR state is calculated within a pure π-type (14,13) active space. The vertical excitation energies in the radicals are calculated within pure or mixed (15,14) active spaces. The geometry optimization of the radicals in the ground D_0 electronic state is performed within the (11,11) active spaces, which are reduced compared to those of (13,13) by excluding two electrons and two orbitals, correlating with the lowest-energy occupied π and highest-energy unoccupied π^{*} orbitals. All reductions are made according to the occupation numbers in the most complete CASSCF active spaces. The orbitals of the largest (18,15) active space are chosen based on the SA(7)-SOSCF(20,16) natural orbitals. Highlighted in red and green are the orbitals that are primarily involved in the electronic transitions discussed in the present work.

SA(7)-CASSCF(14,14*)/(aug*)-cc-pVTZ level of theory. Here, the basis set is also augmented by an additional diffuse function of a p-type with a particularly small exponent (-10) centered at the middle carbon atom. The π^{*} orbital of this highly diffuse shell is included in the pure π-type reduced CASSCF active space and is used to mimic an electron-detachment process. The excluded π orbital refers to a lone pair of electrons predominantly localized at the nitrogen atom of

the amino group of the heterocyclic ring. The ground and electron detached states are included in a state-averaging procedure. A similar method, XMCQDPT2[9]/SA(2)-CASSCF(14,14*)/(p-type d-aug*)-cc-pVTZ has previously been applied to calculate VDE of $HBDI^-$.[10] The larger basis augmented by an additional set of diffuse functions and the state-averaging over the two target states, S_0 and D_0, give a VDE value, which is only 0.1 eV lower than the one obtained in the present work.

Positions of the higher-lying vertical detachment thresholds, D_1 and D_{1n}, are calculated relative to D_0 as vertical excitation energies in the corresponding radical species, using the XMCQDPT2[7]/ SA-CASSCF(15,14)/(aug)-cc-pVTZ method within the pure and mixed active spaces. The higher-lying detachment thresholds lie close to each other; however, their order is sensitive to a particular computational scheme. As a reference, we use the $D_0 \rightarrow D_1$ and $D_0 \rightarrow D_{1n}$ VEEs obtained simultaneously through a single calculation within the mixed active space, using a state-averaging procedure over all target states. We also note, that the slightly different values of the D_1 and D_{1n} VDEs obtained separately, within the different active spaces, do not alter our conclusions.

State-specific ESR gradient calculations of $HBDI^-$ are carried out within a pure π-type active space at the XCMQDPT2[3]/SA(2)-CASSCF(14,13)/(aug)-cc-pVTZ level of theory, where the π active space is reduced by eliminating one occupied orbital with the largest occupation number (Figure S1). The XMCQDPT2 effective Hamiltonian is spanned by the S_0, S_1, and ESR CASSCF wavefunctions, and the state-averaging procedure is over the ground and target ESR states. We note that the order of states here is different, as compared to those of the larger XMCQDPT2 calculations. This is due to the fact that the predominantly two-electron transition (S_2 in the main text) lies higher in energy than the one-electron excitation of the excited shape resonance (S_3 in the main text) at the CASSCF level, and hence the former state is excluded from a perturbative treatment when the XMCQDPT2 effective Hamiltonian is spanned by only three lowest-lying reference CASSCF states. Within a larger reference space of the effective Hamiltonian, the order of states changes when dynamic electron correlation is taken into account. The XMCQDPT2 excited-state gradient is calculated numerically as a two-sided derivative.

Equilibrium geometries of the ground-state radicals are obtained at the MRMP2/SS-CASSCF (11,11)/(aug)-cc-pVTZ level of theory. The active space is here reduced compared to that of (15,14) by excluding four electrons and three orbitals, correlating with the lowest-energy occupied π and highest-energy unoccupied π^* orbitals, as well as with a lone pair of electrons at the nitrogen atom of the heterocyclic ring. For $DMHBDI^-$, the geometry parameters of the ground-state anion and radical have been obtained at the PBE0/(aug)-cc-pVTZ level of theory followed by vibrational analysis, confirming changes in geometry between the anion and the radical.

The temperature-dependent direct $S_0 \rightarrow D_0$ and resonant ESR$\rightarrow D_0$ parts of photoelectron spectra are obtained through a time-domain formalism based on Fourier transforms of the Lax's autocorrelation function within the double harmonic parallel-mode approximation.[11,12] The statistical population of vibrational modes in the ES resonance is assumed upon electron autodetachment. The total propagation time is set to 500 fs, and the autocorrelation function is damped by an exponential function with a time constant of 100 fs. Neither frequency change nor Duschinsky rotation is assumed to occur upon electronic transition. These approximations have previously been validated for $HBDI^-$, and the calculated $S_0 \rightarrow D_0$ spectral profile is in good agreement with the vibrationally resolved photoelectron spectrum measured at 355 nm.[13,14] The vibrational analysis is performed in the anionic ground states, using the MP2/(aug)-cc-pVTZ method. In the case of autodetachment, displacements between two minima are estimated using the quadratic approximation, based on the anion excited-state gradient calculated at the equilibrium geometry of the radical ground state. This approach is validated through calculations of the direct photoelectron spectrum of $HBDI^-$, using various computational schemes, which result in similar photoabsoprtion profiles (see Figure S5). The 328 nm experimental photoelectron spectrum of $HBDI^-$ is fitted using the calculated direct and resonant components, with a shift between their onsets and a width of a convolution function as fitting parameters, which are then used for modeling other spectra in a 355–315 nm range. The shift of 0.22 eV at an excitation wavelength of 328 nm, which corresponds to the calculated vertical excitation energy, is well consistent with the relaxation energy of 0.2 eV in the ESR state, which is estimated within the Lax model, based on the excited-state gradient

calculated at the S_0 equilibrium geometry.

The resonant parts are also calculated based on initial populations of vibrational levels in the ES resonance at a particular excitation wavelength, using a time-independent approach. The instantaneous populations of vibrational levels in the ES (S_3) resonance are defined by the corresponding S_0/S_3 Franck-Condon overlap, and photoelectron spectral shapes of the resonant part are obtained as a product of the S_0/S_3 and S_3/D_0 Franck-Condon overlaps. Stick spectra obtained by direct evaluation of the Franck-Condon integrals are convoluted with a Gaussian function with a half width at half maximum of 13 meV. This parameter is consistent with those used in the Lax model, producing the same spectral shapes for the statistically calculated direct and resonant parts. The original program is used for these calculations.

The direct PD spectrum of DMHBDI$^-$ is obtained using locally estimated displacements, based on the MRMP2/CASSCF(11,11) gradient in D_0 calculated at the MP2 equilibrium geometry of the ground-state anion, since changes in the geometry of DMHBDI upon electron detachment affect both the planar conjugated system and the large-amplitude torsional mode of the methoxy groups. The potential that hinders intramolecular internal rotation of the methoxy groups in D_0 is shallow and highly anharmonic. If taken into account beyond the harmonic approximation, by direct evaluation of the Franck-Condon overlap between the intramolecular rotational eigenfunctions within the real potentials in S_0 and D_0, this low-frequency mode (the harmonic frequency of 75 cm^{-1}(MP2) and 79 cm^{-1}(PBE0) in the anion and 61 cm^{-1}(PBE0) in the radical) will provide additional blurring of the spectral shape. We note that the direct PD spectral shapes of HBDI$^-$ and DFHBDI$^-$ are the same when equilibrium geometries or locally estimated origin shifts are used, thus confirming the validity of the approach for all other in-plane modes well described in the harmonic approximation.

3 Calculated vertical excitation energies in the anions

Table S1: Calculated vertical excitation energies in $HBDI^-$ using different computational schemes within the XMCQDPT2 theory. Highlighted are the bright states with a primarily excited-shape resonance character with respect to autodetachment to D_0. A DFT/PBE0-based one-electron Fock-type matrix is used to obtain energies of all MCSCF semi-canonical orbitals used in perturbation theory. The previous results obtained within a smaller basis set[15] are also shown, with a closed-shell Hartee-Fock-like Fock matrix used to define orbitals and their energies.

	XMCQDPT2[10] SA(7)-CASSCF(16,14)$^{\pi}$ (aug)-cc-pVDZ[15]			XMCQDPT2[7] SA(7)-CASSCF(16,14)$^{\pi}$ (aug)-cc-pVTZ			XMCQDPT2[10] SA(7)-CASSCF(16,14)$^{\pi}$ (aug)-cc-pVTZ		
	eV	nm	[f]	eV	nm	[f]	eV	nm	[f]
**$HBDI^-$**									
$1^1\pi\pi^*$	2.52	492	1.14	2.62	473	1.10	2.63	471	1.17
$2^1\pi\pi^*$	3.71	335	0.00	3.74	331	0.02	3.74	331	0.03
$3^1\pi\pi^*$	**3.82**	**325**	**0.08**	**3.78**	**328**	**0.08**	**3.78**	**328**	**0.06**
$4^1\pi\pi^*$	4.47	278	0.01	4.48	277	0.01	4.49	276	0.01

	XMCQDPT2[9] SA(9)-CASSCF(16,14)$^{\pi/n}$ (aug)-cc-pVDZ[15]			XMCQDPT2[7] SA(3)-CASSCF(16,14)$^{\pi/n}$ (aug)-cc-pVTZ		
	eV	nm	[f]	eV	nm	[f]
**$HBDI^-$**						
$1^1\pi\pi^*$	2.48	501	1.02	2.63	472	1.05
$1^1n\pi^*$	3.50	354	0.00	3.43	362	0.00

Table S2: Calculated vertical excitation energies in $DMHBDI^-$ and $DFHBDI^-$, using different computational schemes within the XMCQDPT2 theory. The shifts in VEEs with respect to $HBDI^-$ are shown in parentheses. Highlighted are the bright states with a primarily excited-shape resonance character with respect to autodetachment to D_0. A DFT/PBE0-based one-electron Fock-type matrix is used to obtain energies of all MCSCF semi-canonical orbitals used in a perturbation theory series.

	XMCQDPT2[7] SA(3)-CASSCF(16,14)$^{\pi/n}$ (aug)-cc-pVTZ				XMCQDPT2[7] SA(7)-CASSCF(16,14)$^{\pi}$ (aug)-cc-pVTZ				XMCQDPT2[7] SA(7)-CASSCF(18,15)$^{\pi}$ (aug)-cc-pVTZ			
	eV	(shift)	nm	[f]	eV	(shift)	nm	[f]	eV	(shift)	nm	[f]
**$DMHBDI^-$**												
$1^1\pi\pi^*$	2.57	(−0.06)	482	1.00	2.53	(−0.09)	491	1.05	2.46	(−0.16)	504	1.04
$1^1n\pi^*$	3.59	(+0.16)	345	0.00	–		–	–	–		–	–
$2^1\pi\pi^*$	–		–	–	3.64	(−0.10)	341	0.00	3.60	(−0.14)	345	0.00
$3^1\pi\pi^*$	–		–	–	3.93	(−0.55)	316	0.02	3.79	(−0.69)	328	0.00
$4^1\pi\pi^*$	–		–	–	**4.18**	**(+0.40)**	**297**	**0.08**	**4.08**	**(+0.30)**	**304**	**0.10**
**$DFHBDI^-$**												
$1^1\pi\pi^*$	2.62	(−0.01)	474	1.04	2.58	(−0.04)	480	1.07	2.53	(−0.09)	491	1.06
$1^1n\pi^*$	3.62	(+0.19)	342	0.00	–		–	–	–		–	–
$2^1\pi\pi^*$	–		–	–	3.64	(−0.10)	341	0.00	3.58	(−0.16)	346	0.00
$3^1\pi\pi^*$	–		–	–	**4.07**	**(+0.29)**	**305**	**0.05**	4.02	(−0.46)	308	0.02
$4^1\pi\pi^*$	–		–	–	**4.34**	**(−0.14)**	**286**	**0.05**	**4.20**	**(+0.42)**	**295**	**0.07**

4 Calculated higher-lying vertical detachment thresholds

The higher-lying vertical detachment thresholds, D_1 and D_{1n}, are calculated as a sum of the electron detachment energy, $S_0 \rightarrow D_0$, and the vertical excitation energies in the radical species at the equilibrium geometries of the ground-state anions (Table S3). Here, the use of mixed active spaces with a simultaneous averaging over the target states D_0, D_{1n}, and D_1 consistently yields the D_{1n} state lying below D_1 for all the chromophores.

Table S3: Calculated vertical excitation energies (eV) in the neutral radicals HBDI, DMHBDI, and DFHBDI with respect to D_0 at the equilibrium geometries of the ground-state anions. The vertical detachment energies with respect to the ground-state of the anions are shown in parenthesis. Different computational schemes at the XMCQDPT2[7]/SA-CASSCF(15,14)/(aug)-cc-pVTZ level of theory are used within pure π and mixed π/n active spaces shown in Figure S1. Energies of all MCSCF semi-canonical orbitals (OE) used in a perturbation theory series are obtained using both DFT/PBE0-based and closed-shell Hartree-Fock-like Fock matrices.

	**DMHBDI**		**HBDI**		**DFHBDI**	
	D_{1n}	D_1	D_{1n}	D_1	D_{1n}	D_1
simultaneous calculations						
XMCQDPT2[7: DFT-OE]/	1.36		1.23		1.48	
SA(7)-CASSCF(15,14)$^{\pi/n}$	(4.05)		(3.97)		(4.47)	
		1.66		1.66		1.67
		(4.35)		(4.40)		(4.66)
XMCQDPT2[7: HF-OE]/	1.47		1.30		1.52	
SA(7)-CASSCF(15,14)$^{\pi/n}$	(4.16)		(4.04)		(4.51)	
		1.63		1.62		1.62
		(4.32)		(4.36)		(4.61)
separate calculations						
XMCQDPT2[7: DFT-OE]/	1.60		1.42		1.65	
SA(2)-CASSCF(15,14)$^{\pi/n}$	(4.29)		(4.16)		(4.64)	
		–		–		–
XMCQDPT2[7: DFT-OE]/	–		–		–	
SA(2)-CASSCF(15,14)$^{\pi}$		1.43		1.43		1.42
		(4.12)		(4.17)		(4.41)
XMCQDPT2[7: DFT-OE]/	–		–		–	
SA(7)-CASSCF(15,14)$^{\pi}$		1.46		1.46		1.47
		(4.15)		(4.20)		(4.46)

5 Character of the electronic transitions

The XMCQDPT2 zeroth-order natural orbitals primarily involved in the $S_0 \rightarrow S_i$ ($i = 1-4, 1n$) transitions in $HBDI^-$, $DMHBDI^-$, and $DFHBDI^-$ are shown in Figures S2 and S3. We note that the order of the excited states slightly changes upon introducing the substituents to the phenoxide group of $HBDI^-$, thus resulting in a different pattern of mixing between the higher-lying states. In $HBDI^-$, the dark $2^1\pi\pi^*$ state with a large fraction of the two-electron $\pi_{HOMO} \rightarrow \pi^*_{LUMO}$ excitation lies very close in energy to the $3^1\pi\pi^*$ excited shape resonance, which corresponds to the one-electron $\pi_{HOMO} \rightarrow \pi^*_{Ph}$ excitation with an appreciable oscillator strength. These states are mixed to some extent, and the mixing depends on a particular computational scheme. By increasing the number of CASCI reference states, which are allowed to interact through an effective Hamiltonian calculated perturbatively, a slightly larger mixing is observed. In particular, the XMCQDPT2[7]/CASSCF(16,14) yields more localized states with oscillator strengths of 0.02 and 0.08 for the $S_0 \rightarrow S_2$ and $S_0 \rightarrow S_3$ transitions, respectively, whereas these transitions with oscillator strengths of 0.03 and 0.07 are mixed to a larger extent at the XMCQDPT2[10]/CASSCF(16,14) level (see Figure S2). Nevertheless, both computational schemes give the same vertical excitation energies, 331 nm (3.74 eV) and 328 nm (3.78 eV), for the $2^1\pi\pi^*$ and $3^1\pi\pi^*$ states, respectively, and the latter state has a predominant ESR character (Table S1).

In the substituted chromophore anions, the ESR states lie considerably higher in energy, and these states become mixed with their higher-lying counterparts – excited states, which correspond to the one-electron $\pi_{Ph} \rightarrow \pi^*_{LUMO}$ excitation. The π_{Ph} and π^*_{Ph} orbitals, which are highly localized on the phenoxide group, are largely affected by the π electron donating substituents introduced into the ring, leading to the S_0–S_3 and S_0–S_4 energy gaps increasing and decreasing, respectively, as compared to those in $HBDI^-$. The order of these states therefore changes in the substituted chromophore anions, and the $4^1\pi\pi^*$ state becomes the ESR state.

In $DFHBDI^-$ and $DMHBDI^-$, the mixing between the $3^1\pi\pi^*$ and $4^1\pi\pi^*$ states also depends on the computational scheme, in particular, on the choice of the active space. Here, we have considered two variants of the pure π-type active spaces. The first one is the most complete pure

π-type valence space of HBDI$^-$, (16,14), and the second one is further enlarged by including an additional molecular orbital, which corresponds to a linear combination of the doubly occupied π orbitals of the substituents, (18,15) (see Figure S1). The orbitals localized on the substituents also have a noticeable contribution to those molecular orbitals that are primarily involved in the transition to the ESR state. By enlarging the CASSCF active space, the π electron donating effects of the substituents can be probed directly under the influence of both static and dynamic electron correlation.

In the substituted chromophore anions, the $3^1\pi\pi^*$ and $4^1\pi\pi^*$ excited states calculated at the XMCQDPT2[7]/SA(7)-CASSCF(18,15) level of theory are more localized in terms of their expansions through configurational state functions compared to those obtained within the XMCQDPT2[7]/ SA(7)-CASSCF(16,14) approach. In particular, the leading configurations of these states in DMHBDI$^-$ have weights of 0.4 ($\pi_{Ph} \rightarrow \pi^*_{LUMO}$) and 0.5 ($\pi_{HOMO} \rightarrow \pi^*_{Ph}$), respectively, whereas the corresponding counterparts make much smaller contributions of 0.1 ($\pi_{HOMO} \rightarrow \pi^*_{Ph}$) and 0.05 ($\pi_{Ph} \rightarrow \pi^*_{LUMO}$) to these states, respectively. Accordingly, the $4^1\pi\pi^*$ state has a pronounced ESR character, and the $S_0 \rightarrow S_4$ transition with an oscillator strength of 0.10 is, therefore, optically much brighter than the $S_0 \rightarrow S_3$ transition (see Table S2 and Figure S3). In contrast to this, the calculations within the smaller active space yield almost equal contributions of $\sim$0.3 from both configurations to each state. Furthermore, the reference CASSCF(16,14) states strongly interact under the influence of dynamic electron correlation via an effective Hamiltonian calculated perturbatively, thus indicating the importance of the π orbitals of the substituents for a better description of the unperturbed states. The XMCQDPT2[7]/SA(7)-CASSCF(16,14) VEEs equal 315 nm (3.94 eV) and 297 nm (4.18 eV) for the $S_0 \rightarrow S_3$ and $S_0 \rightarrow S_4$ transitions, respectively, with the corresponding oscillator strengths of 0.02 and 0.08.

In DFHBDI, there is a larger mixing of the $3^1\pi\pi^*$ and $4^1\pi\pi^*$ excited states at all levels of theory. The XMCQDPT2 calculations within the smaller active space give VEEs of 304 nm (4.08 eV) and 286 nm (4.34 eV) with an oscillator strength of 0.05 for both transitions (Table S2). The larger XMCQDPT2 calculations provide almost equal contributions of $\sim$0.3 from both configurations to

each state, however, the transition to the $4^1\pi\pi^*$ state located at 295 nm (4.20 eV) becomes brighter with an oscillator strength of 0.07, whereas the lower-lying state at 308 nm (4.02 eV) becomes nearly dark with an oscillator strength of 0.02.

At all levels of theory, the ESR state lies higher in energy in the substituted chromophore anions, as compared to that of $HBDI^-$. Therefore, this state cannot be populated significantly upon excitation at 328 nm, in contrast to $HBDI^-$, where it is located at 328 nm. Moreover, if populated, the ESR state should appear on the low eBE side of the 328 nm photoelectron spectra of the biomimetic GFP chromophores, and it does not contribute to a considerable broadening at the high eBE side of the photoelectron distributions (see also the main text).

The low-lying $1^1\pi\pi^*$ and $1^1n\pi^*$ states are well described by the corresponding single configurations (see Figure S2). The $1^1\pi\pi^*$ states in all anions are electronically bound, whereas the $1^1n\pi^*$ states lie above the D_0 electron detachment thresholds. The XMCQDPT2 zeroth-order natural orbitals of the D_0, D_{1n}, and D_1 states in $HBDI^-$, $DMHBDI^-$, and $DFHBDI^-$ are shown in Figure S4. The D_0 electron continuum correlates with the electronic configuration of the excited shape resonance, $\pi_{HOMO} \rightarrow \pi^*_{Ph}$, which is the leading configuration in the $3^1\pi\pi^*$ and $4^1\pi\pi^*$ states in $HBDI^-$ and the substituted anions, respectively. The D_{1n} and D_1 electron detachment thresholds primarily correlate with the electronic configurations of the $1^1n\pi^*$ and $2^1\pi\pi^*$ resonances, respectively, and autodetachment out these states to the corresponding continua is a one-electron process. It is worth noting, that the $1^1n\pi^*$ and $2^1\pi\pi^*$ states, which are Feshbach resonances with respect to D_0, lie below the higher-lying detachment thresholds, with respect to which they become of a shape type.

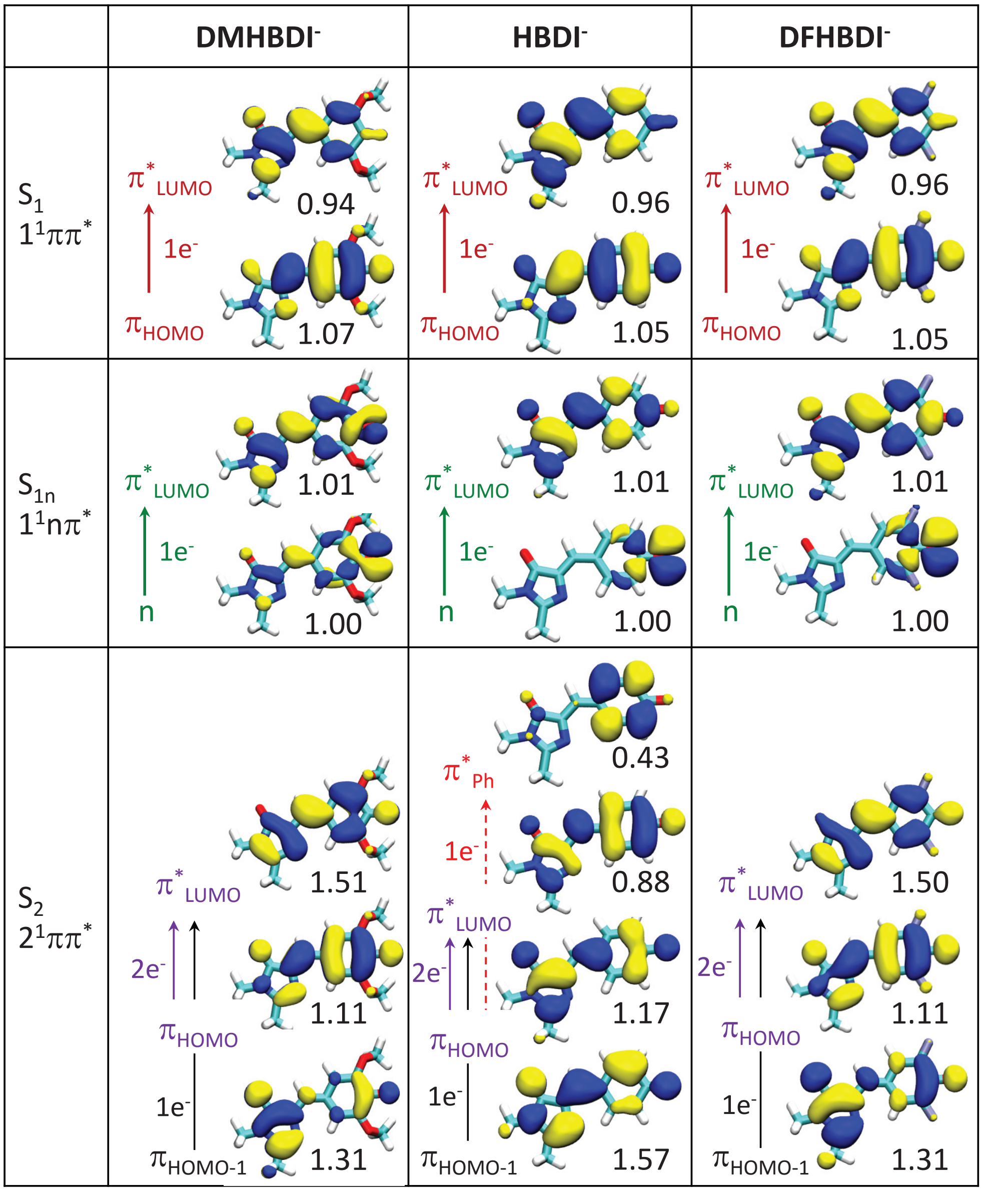

Figure S2: The XMCQDPT2 zeroth-order natural orbitals of the S_1, S_{1n}, and S_2 excited states in the deprotonated chromophores. Shown are the orbitals, which are partially filled, with the occupation numbers larger than 0.2 and less than 1.8. For clarity, the natural orbitals are also correlated with the canonical ones. The leading configurations are depicted as the corresponding one- and two-electron transitions. Note that the natural orbitals of particular states can be rotated within the active space. The largest active spaces and the largest reference spaces considered in the present work are used to illustrate the character of the corresponding electronic transitions.

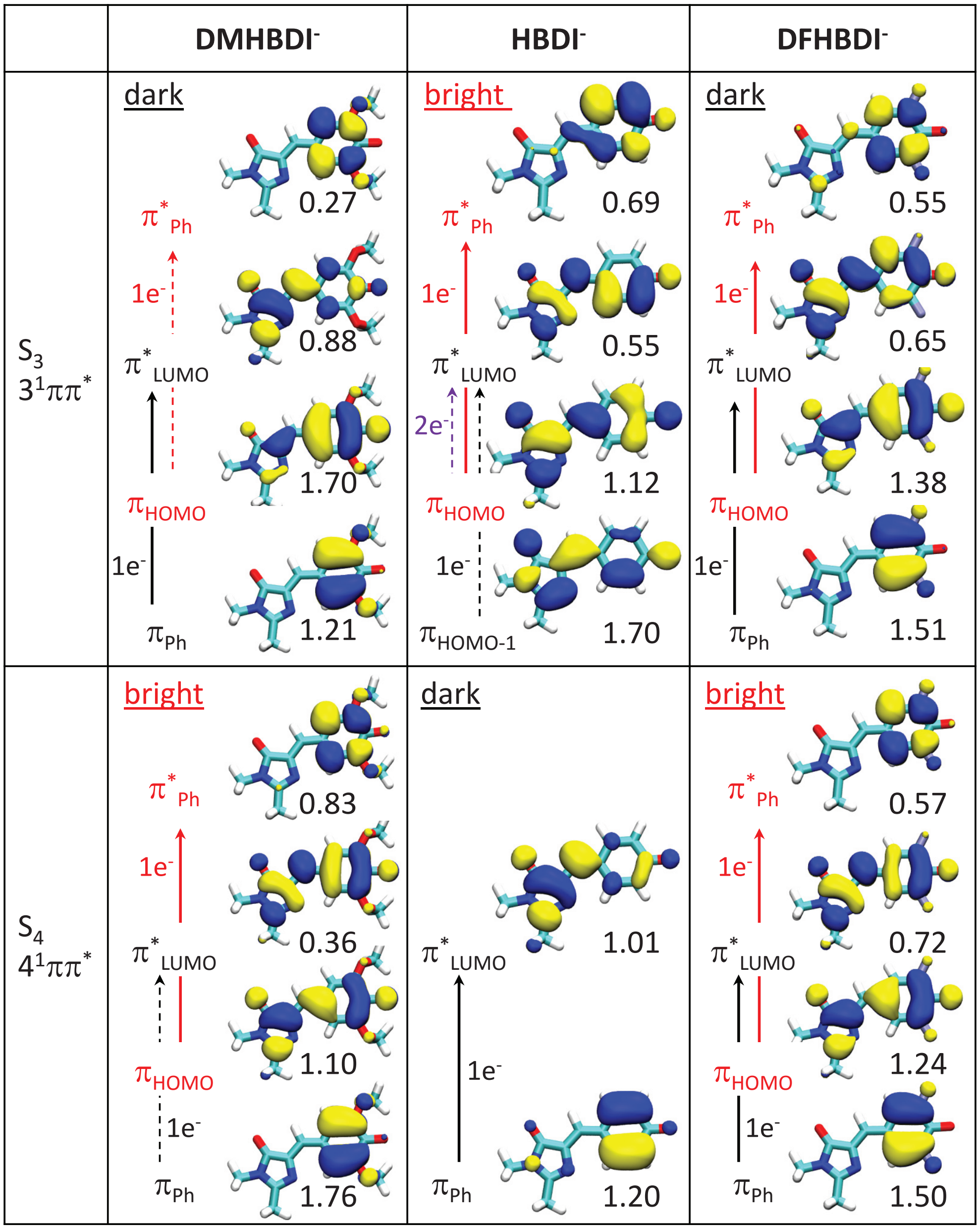

Figure S3: The XMCQDPT2 zeroth-order natural orbitals of the S_3 and S_4 excited states in the deprotonated chromophores. Shown are the orbitals, which are partially filled, with the occupation numbers larger than 0.2 and less than 1.8. For clarity, the natural orbitals are also correlated with the canonical ones. The leading configurations are depicted as the corresponding one- and two-electron transitions. The resonances are also labeled according to the transition dipole moments from S_0 (bright *vs.* dark). Note that the natural orbitals of particular states can be rotated within the active space. The largest active spaces and the largest reference spaces considered in the present work are used to illustrate the character of the corresponding electronic transitions.

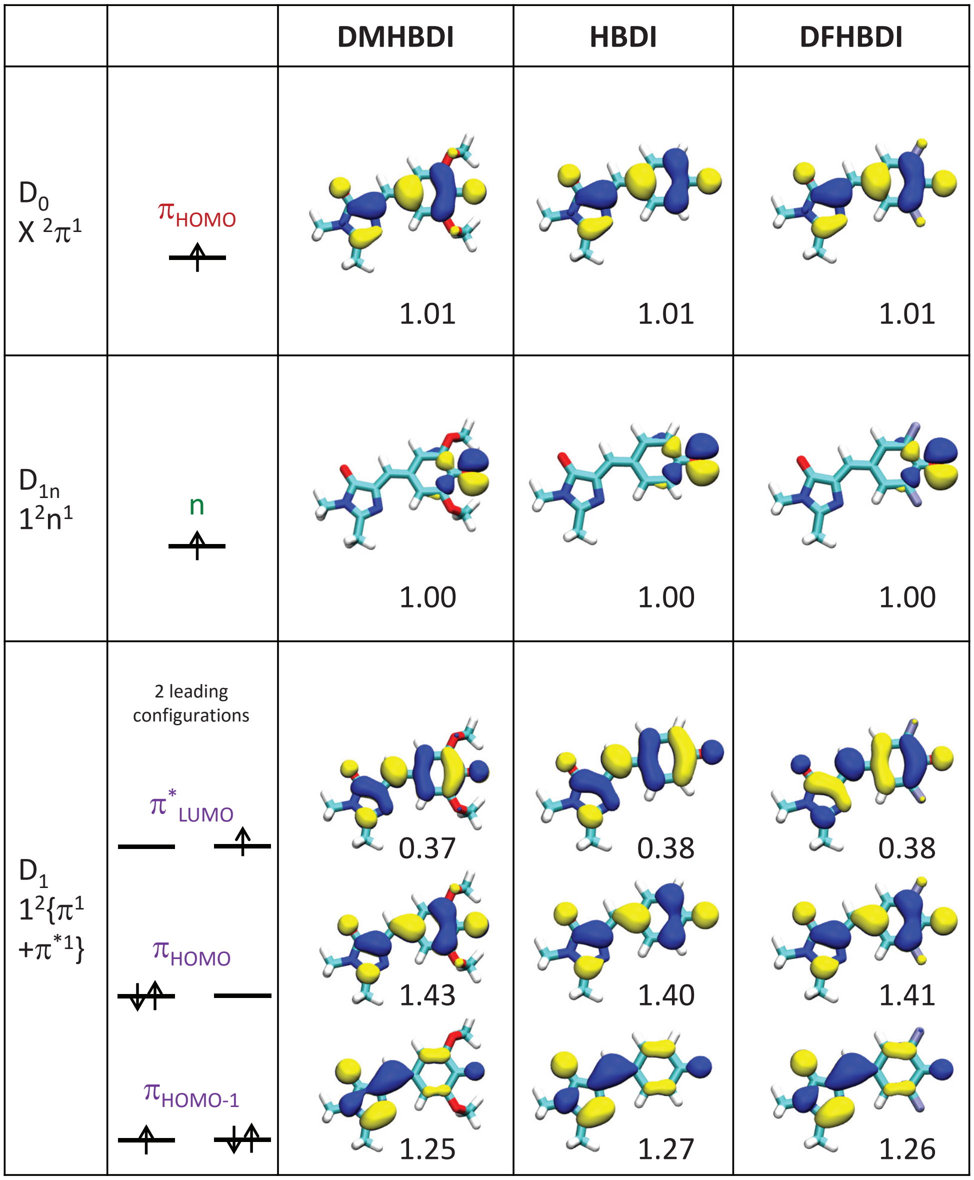

Figure S4: The XMCQDPT2 zeroth-order natural orbitals of the D_0, D_{1n}, and D_1 states in the radical chromophores. Shown are the orbitals, which are partially filled, with the occupation numbers larger than 0.3 and less than 1.7. For clarity, the natural orbitals are also correlated with the canonical ones. Note that the natural orbitals of particular states can be rotated within the active space. The XMCQDPT2[7]/SA(7)-CASSCF(15,14) calculations with the pure π-type active space is used to illustrate the character of the D_0 and D_1 states, whereas the singly occupied n orbital of the D_{1n} state is from the calculations within the mixed active space.

6 $S_0 \rightarrow D_0$ photoelectron spectral shape in PD from HBDI$^-$

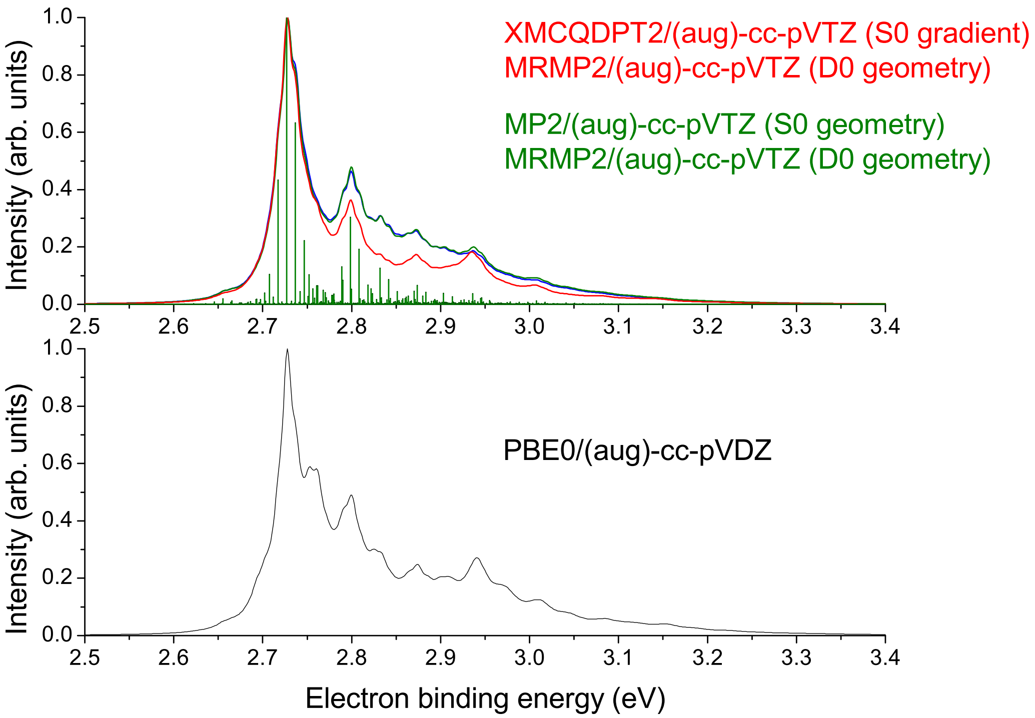

Figure S5: Calculated $S_0 \rightarrow D_0$ spectral profiles at 300 K using different computational schemes. Shown are the spectra calculated using the MP2/(aug)-cc-pVTZ (S_0) and MRMP2/CASSCF(11,11)/(aug)-cc-pVTZ (D_0) equilibrium geometries of the ground-state anion and the radical, respectively, (green, upper panel), as well as the PBE0/(aug)-cc-pVDZ geometries obtained previously[13] (lower panel). The stick spectrum based on the Franck-Condon factors calculated through a time-independent approach at 300 K is also shown. The spectral profiles obtained using the S_0-to-D_0 origin shifts that are estimated within the quadratic approximation based on either the MRMP2/CASSCF(11,11) gradient in D_0 calculated at the MP2 geometry of the S_0 anion (blue, coinciding with the green spectrum) or the XMCQDPT2/CASSCF(14,13) gradient in S_0 calculated at the MRMP2 geometry of the D_0 radical (red) are depicted in the upper panel. The latter computational scheme is also used for simulating a spectral profile of the resonant part of the HBDI$^-$ photoelectron spectra.

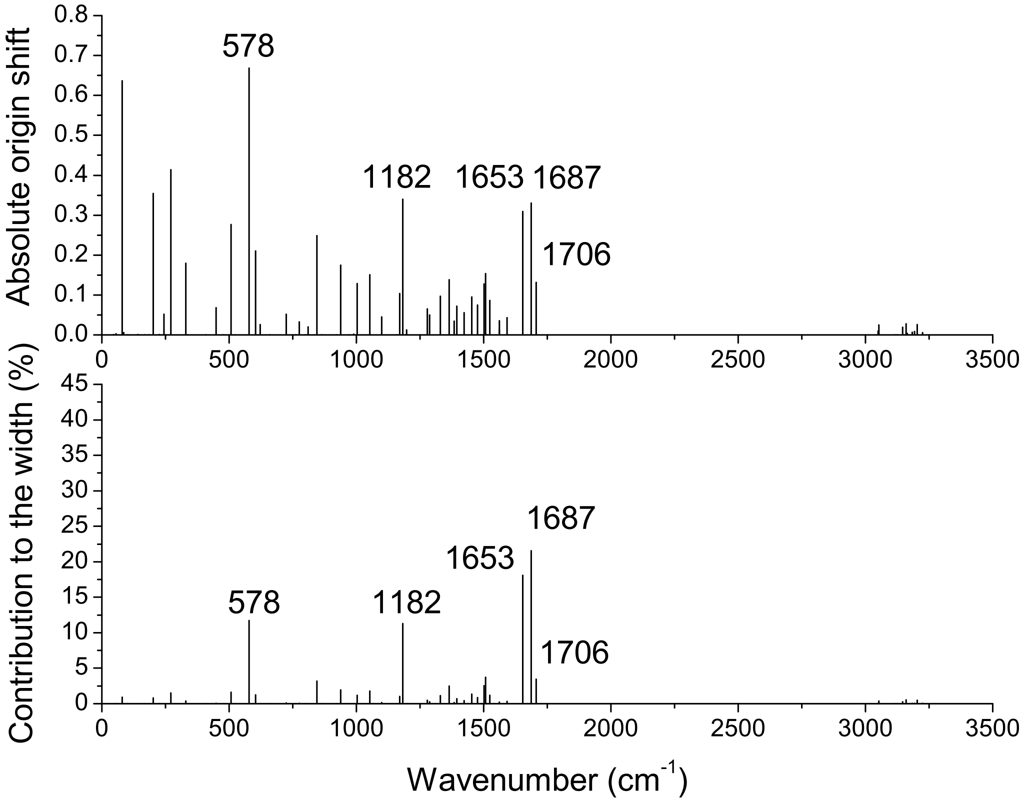

Figure S6: Franck-Condon active modes in direct photodetachment from $HBDI^{-}$. Shown are absolute values of the S_0/D_0 dimensionless origin shifts along each normal mode, *i.e.* the S_0-to-D_0 minimum displacements along each normal mode in the dimensionless normal coordinates (multiplied by $\sqrt{\omega/\hbar}$), (upper panel) and contributions from each normal mode to the overall spectral width calculated as a Gaussian dispersion at 300 K (lower panel, see eq. 1 below). The most active normal modes are depicted in Figure S9, except for the 578 cm^{-1} mode, which corresponds to a valence angle bending mode predominantly localized on the heterocyclic ring. Harmonic MP2/(aug)-cc-pVTZ frequencies calculated in the ground electronic state of the anion are used for the analysis, and the origin shifts are obtained based on the XMCQDPT2/CASSCF(14,13)/(aug)-cc-pVTZ S_0 gradient calculated at the MRMP2/CASSCF(11,11)/(aug)-cc-pVTZ equilibrium geometry of the D_0 radical. The corresponding spectral shape is shown in red in Figure S5. Note that the calculated origin shifts (less than $\sqrt{2}$) indicate that the 0–0 transition has the maximum Franck-Condon strength.

7 Autodetachment out of S_1 above the S_1/D_0 crossing

The calculated $S_0 \rightarrow D_0$ spectral profiles at 20 K and 300 K (Figures S7a and S7b) are consistent with those obtained experimentally at a photon energy of 355 nm,[14] apart from an additional broadening at the higher eBE part and two additional features located at ∼2.9 (broad) and 3.1 eV in the experimental spectra. These features are most prominent in the vibrationally resolved spectrum taken at 20 K. The peak at 3.1 eV is located far apart with respect to ADE (2.73 eV) and can not be reproduced by any calculations of the S_0/D_0 spectral shape (see, for example, the spectra obtained using various computational schemes in Figure S5). By increasing a simulation temperature, a density of vibronic transitions can effectively be increased (note the progressively broader and blurred spectral shapes calculated at 20, 300, and 500 K in Figure S7a). The peak at 3.1 eV can not be accounted for at any temperature. Furthermore, hot transitions at higher temperatures result in significantly broader photoelectron distributions at the low eBE part compared to that measured experimentally. The calculated profiles at 20 and 300 K reproduce the onsets in the experimental spectra, thus indicating that the experimental conditions are indeed close to these temperatures, and there is no any substantial heating of gas-phase ions prior their photoexcitation.

The peak at 3.1 eV and the additional broadening of the experimental spectrum at the high eBE part registered even at a very low temperature are remarkable, since this suggests that more vibrational transitions are involved compared to those defined by the calculated $S_0 \rightarrow D_0$ Franck-Condon factors. These additional transitions result from a different electron emission mechanism. Given that the additional features are progressively shifted by ∼0.2 and 0.4 eV with respect to the direct detachment threshold, they can be assigned to vibrational autodetachment out the S_1 state above the S_1/D_0 crossing. The presence of a group of high-frequency stretching modes with a ∼0.2 eV energy, which are active in autodetachment out of S_1,[10] indeed supports this. The lowest-lying crossing point between S_1 and D_0 is reached along these modes, where the most active one is the 1706 cm^{-1} mode (experimental frequency of 1660 cm^{-1},[16] see Figure S9). We note that autodetachment out of the S_1 state above the S_1/D_0 crossing is a one-electron process and should therefore proceed efficiently.

We assign the peaks at $\sim$2.9 and 3.1 eV to vibrational autodetachment from S_1, where a high excitation level above the S_1/D_0 crossing is reached for a particular emission-active high-frequency mode with an energy of $\sim$0.2 eV. A number of allowed vibronic transitions from S_1 to D_0 is limited by the fact that D_0 lies higher in energy than S_1. Therefore, only transitions from higher vibrational levels of the anion to lower vibrational levels of the radical can be operative in autodetachment from S_1. In Figure S7c, the transitions from the 5th level, which corresponds to a photoexcitation energy at 355 nm, are illustrated. Remarkably, a high initial excitation level can significantly alter a distribution of Franck-Condon strengths in $I^2_{n\to n'}$ for each n. The squares of the Franck-Condon overlap integrals within $I^2_{5\to n'}$ are shown as a function of absolute origin shifts in Figure S7d. The transitions $5 \to 3$ and $5 \to 2$ gain considerable strengths for origin shifts greater than 0.8. Indeed, the absolute value of the S_1/D_0 origin shift along the 1706 cm^{-1} mode has previously been calculated by us to be close to 0.8.[10] The Franck-Condon strength for the $5 \to 2$ transition reaches a value of $\sim$0.05, and the relative height of the peak at 3.1 eV, shifted by 0.4 eV (2×0.2) from a true eBE at 2.73 eV, matches this intensity. The $5 \to 3$ transition is located at $\sim$2.9 eV, which is 0.2 eV apart from the origin of the direct transition and has a larger Franck-Condon strength of 0.2. An additional spectral blurring even at low temperatures is expected due to a finite lifetime of these highly excited vibrational levels in S_1.

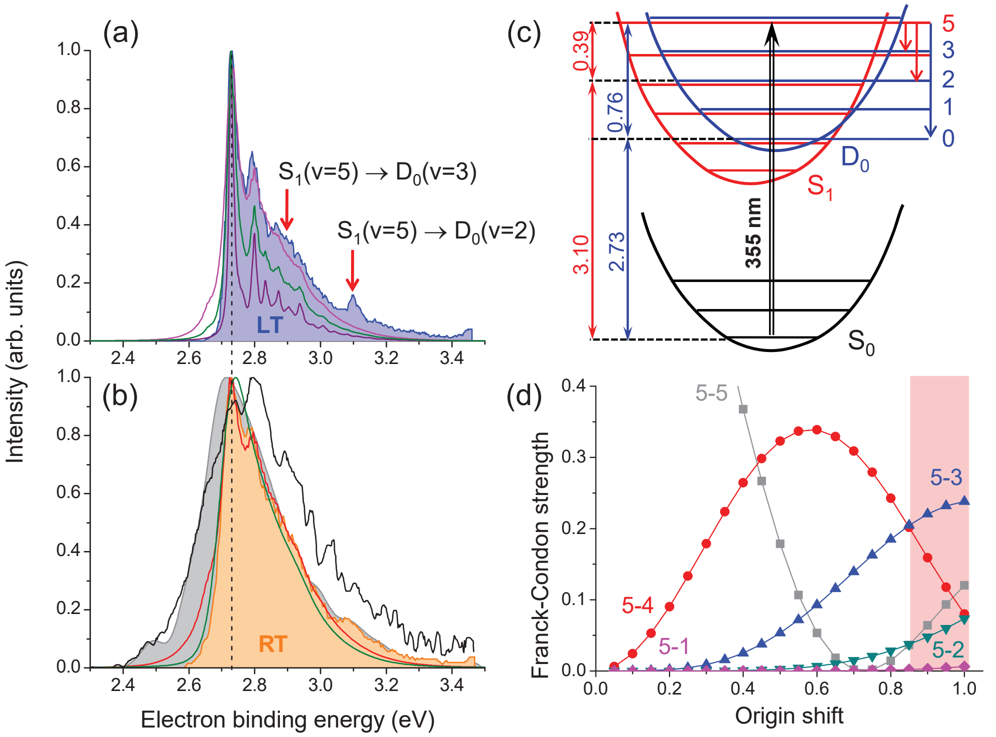

Figure S7: Photodetachment from $HBDI^-$ at 355 nm. Shown are the experimental photoelectron spectra plotted as a function of eBE at 20 K (a) and 300 K (b) as the shaded blue,[14] orange,[14] and gray[13] areas, as well as the black curve.[17] The calculated spectral profiles of the direct $S_0 \to D_0$ transition are depicted at 20 K (purple), 300 K (green), and 500 K (magenta) in panel (a), as well as at 300 K, which is also convoluted with a Gaussian function of a 39 meV HWHM, (green) and 800 K (red) in panel (b). Note that a higher density of vibronic transitions gained through either higher simulation temperatures or through an effective additional broadening (shown at 300 K) do not reproduce all the features in the experimental shapes, in particular, a peak at 3.1 eV, which is shifted by 0.37 eV from a true BE shown as the black dotted line. The mechanism of autodetachment above the S_1/D_0 crossing from the S_1 state is illustrated in panel (c). Three harmonic potential energy surfaces are schematically shown along a Franck-Condon active normal mode with an energy of 0.2 eV. A group of four stretching modes of ∼1600–1700 cm^{-1} has indeed been found among the active modes in both the S_0–S_1 and S_1–D_0 transitions.[10] The Franck-Condon strengths $I^2_{5\to n'}$ for $n' = 1-5$ plotted as a function of origin shifts bewteen the two minima in S_1 and D_0 are shown in panel (d). Note that the 5→3 transition has the largest strength, whereas the 5→5 and 5→4 transitions can not occur upon vibrational autodetachment above the S_1/D_0 crossing.

8 ESR→D_0 photoelectron spectral shape in PD from $HBDI^-$

Since the excited shape resonance has a lifetime of ∼55 fs,[17] internal vibrational energy redistribution (IVR) in the ESR state is not complete, and autodetachment is influenced by vibrational dynamics in the resonance state and vibrational energy transfer between various modes. The population of vibrational levels in the resonance state is non-statistical and changes in time. Therefore, the statistical calculation of the ESR/D_0 Franck-Condon overlap, where the ground vibrational state for each mode is mostly populated, does not represent the entire spectral profile of the resonant part. The non-statistical spectrum is expected to be broader due to a finite lifetime of each excited vibrational level in the ESR state, as well as due to a different ESR/D_0 Franck-Condon overlap $I^2_{n\to n';n\neq 0}$ for transitions from excited vibrational levels, as compared to the strength of $I^2_{0\to n'}$ for transitions from the ground vibrational state (see Figure S8). The higher initial excitation level n of a particular mode, the broader is the resulting Franck-Condon envelope. However, when the initial excitation level is not so high, the statistically calculated spectrum should peak close to the maximum of the non-statistical one. In other words, if the $0 \to 0$ transition is a leading term of $I^2_{0\to n'}$, then the $n \to n$ transition also bears the maximum strength of $I^2_{n\to n'}$ for small n. The highest possible value of n that does not alter a leading term of the Franck-Condon envelope depends on the ESR/D_0 origin shift (Figure S8).

The calculated ESR/D_0 origin shift in $HBDI^-$, which predominantly defines the overall spectral width, equals to 0.72 in the dimensionless coordinates of the 1687 cm^{-1} normal mode. Both the $0 \to 0$ and $1 \to 1$ transitions are the leading terms of the corresponding $I^2_{0\to n'}$ and $I^2_{1\to n'}$ Franck-Condon envelopes and equal to 0.77 and 0.42, respectively. For the higher excitation level, $n = 2$, the leading term shifts from the $2 \to 2$ transition ($I^2_{2\to 2} = 0.20$) to the $2 \to 3$ ($I^2_{2\to 3} = 0.34$) and $2 \to 1$ ($I^2_{2\to 1} = 0.30$) transitions (see Figure S8 for $\Delta = 0.7$). Upon resonant excitation of the ESR state at 328 nm that coincides with the calculated VEE of the $S_0\to S_3$ transition, only the ground and first excited vibrational levels are expected to be appreciably populated. The estimated relaxation energy in the ESR (S_3) state is 0.22 eV, which supports a not so high vibrational excitation level of the most Franck-Condon active mode in autodetachment. Therefore, the ESR/D_0 resonant part

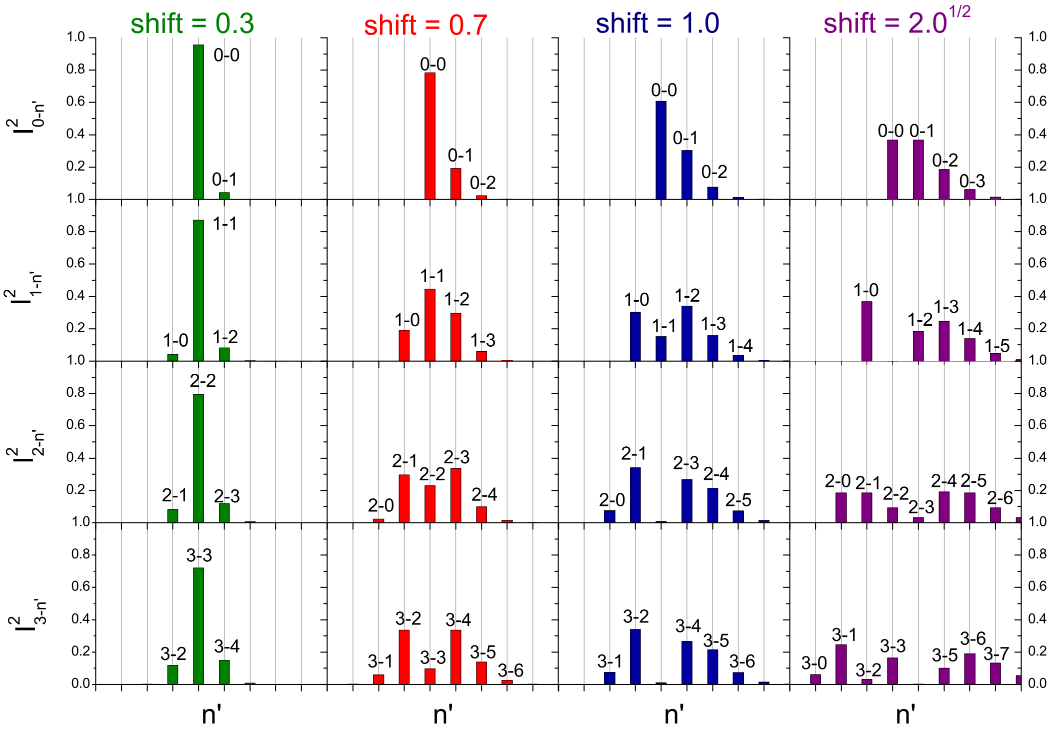

Figure S8: Squares of the Franck-Condon overlap integrals, $I^2_{0\to n'}$, $I^2_{1\to n'}$, $I^2_{2\to n'}$, and $I^2_{3\to n'}$, as a function of n'. Shown are the Franck-Condon envelopes for $|\Delta| = 0.3, 0.7, 1.0, \sqrt{2}$. Note that the overall intensity within a particular Franck-Condon envelope, $\sum_{n'} I^2_{n\to n'} = 1$, becomes diluted over an increasingly larger number of transitions upon increasing initial excitation level. A leading term in the Franck-Condon envelope stays the same for small n (at least for $n = 0$ and $n = 1$), when origin shifts are relatively small (≤ 0.8). The 0.7 origin shift corresponds to the absolute value of the calculated ESR/D_0 minimum displacement along the most Franck-Condon active high-frequency mode of 1687 cm^{-1}. Based on the estimated S_0/ESR geometry relaxation energy, only the ground and to a lesser extent first excited vibrational levels are expected to be populated in the ESR state upon resonant photoexcitation, thus the resonant part of the HBDI$^-$ photoelectron spectrum primarily contains contributions from $I^2_{0\to n'}$ and $I^2_{1\to n'}$ ($\Delta = 0.7$). The statistically calculated resonant part is predominantly defined by $I^2_{0\to n'}$.

peaks at the same position as the statistically calculated eBE distribution.

At the same time, the non-statistical eBE distribution is broader due to additional transitions that occur from excited vibrational levels with non-negligible oscillator strengths, *e.g.* the $1 \to 0$ transition has the strength of $I^2_{1\to 0} = 0.2$ for $\Delta = 0.7$ (see Figure S8). For the 1687 cm^{-1} (0.21 eV) mode, the $1 \to 0$ transition of the resonant part is located at 2.74 eV in the eBE distribution, which is very close to the origin of the direct $S_0 \to D_0$ transition. This provides an additional

source for the spectral blurring also for the direct part (*i.e.* within the energy domain of the direct part). In our calculations, these additional vibronic transitions occurring from excited vibrational levels populated upon photoexcitation, as well as a nuclear lifetime broadening (*i.e.* vibrational decoherence) in the resonance state are effectively taken into account by convoluting both the direct and resonant parts calculated statistically with a Gaussian function with a HWHM of 0.1 eV. The same parameters are used in convoluting spectral shapes of the photoelectron spectra of $HBDI^-$ at various excitation wavelengths within the entire S_0–S_3 absorption band.

The statistically calculated resonant part of the photoelectron spectrum of $HBDI^-$, which is defined by the overlap of vibrational wave functions between the anionic excited shape resonance and the neutral ground state, is shown in Figure 2 of the main text. Absolute values of ESR-to-D_0 minimum displacements (origin shifts, Δ) along each Franck-Condon mode in the dimensionless normal coordinates (multiplied by $\sqrt{\omega/\hbar}$) are shown in Figure S9. The contributions from each normal mode to the overall band width are estimated based on the short-time approximation of the Lax's autocorrelation function that produces a nearly Gaussian absorption band.[12] The Gaussian variance parameter accounts for temperature, origin shifts Q_j, and frequencies ν_j:

$$(\delta\nu)^2 = \sum_j (Q_j^2/2)(\nu_j)^2(2\bar{n}_j + 1), \tag{1}$$

where $\bar{n}_j$ is the thermal average occupation number of the j-th mode. It is worth noting that the major contribution to the width comes from high-frequency modes, while those with low frequencies promote a spectral blurring. All active Franck-Condon modes refer to in-plane vibrations of the conjugated part of the chromophore anion. Upon autodetachment out of the ESR state, the $\pi^1_{HOMO}\pi^{*1}_{Ph} \rightarrow \pi^1_{HOMO} + e^-$ transition mostly affects the phenoxide moiety of $HBDI^-$; however, a number of normal modes delocalized over the entire π conjugated system respond to the changes in the electron density redistribution. At the same time, the origin shifts are not very large along all the modes, and the 0–0 transition has the largest Franck-Condon strength. The most prominent feature of the calculated spectral shape of the resonant part is a shoulder located ~0.21 eV

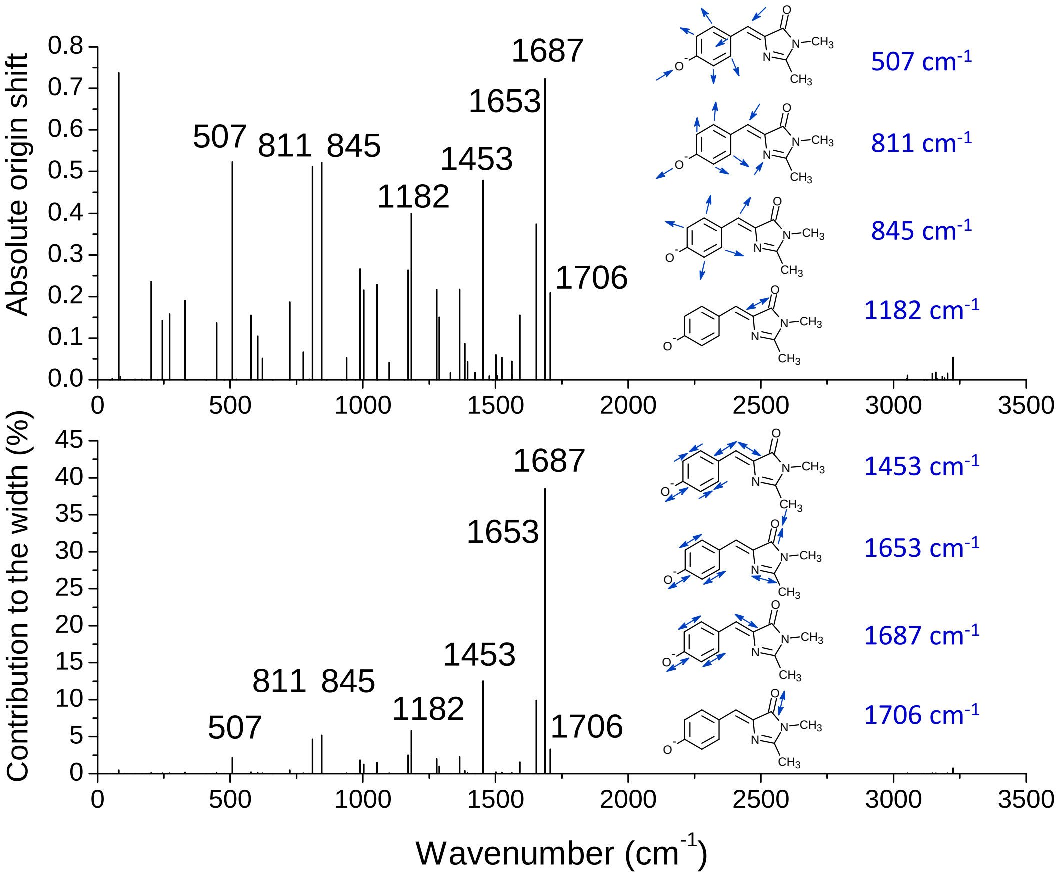

Figure S9: Franck-Condon active modes in autodetachment from the excited shape resonance in $HBDI^-$. Shown are absolute values of the origin shifts between the anionic excited and radical ground states along each normal mode (upper panel) and contributions from each normal mode to the overall spectral width calculated as a Gaussian dispersion at 300 K (lower panel). The most active normal modes are depicted on the right. Harmonic MP2/(aug)-cc-pVTZ frequencies calculated in the ground electronic state of the anion are used for the analysis, and the origin shifts are obtained based on the XMCQDPT2/CASSCF(14,13)/(aug)-cc-pVTZ ESR gradient calculated at the MRMP2/CASSCF(11,11)/(aug)-cc-pVTZ equilibrium geometry of the D_0 radical. Note that the calculated origin shifts (less than $\sqrt{2}$) indicate that the 0–0 transition has the maximum Franck-Condon strength.

apart from the most intense 0–0 transition, which refers to the 0–1 excitation of the stretching mode with a frequency of 1687 cm^{-1}. The frequency of this mode calculated previously at the PBE0/(aug)-cc-pVDZ level of theory is 1700 cm^{-1}.[10]

9 Variations in experimental and theoretical spectra

Figure 4 of the main text shows that the experimental photoelectron spectra measured at 355–315 nm by various groups are perceptibly different. This can be rationalized by taking into account an interplay between the direct and resonant PD channels, which defines the overall spectral shape. The interplay is sensitive to experimental conditions, and, in particular, to the temperature of the ground-state anions. A slightly different ratio between the two channels may result in a somewhat different skewness of the spectral shape. This is, for example, seen in the experimental photoelectron spectra at 315 nm, which can be fitted with a slightly different ratio between the two parts, for example, of 1.4 (orange curve[5]) and 1.1 (black curve[17]) (see Figure S10). It is even more evident at excitation energies below the adiabatic $S_0 \rightarrow S_3$ transition, *e.g.* at 355 and 350 nm, where resonant PD via the ESR state is operative only if higher vibrational levels are substantially populated in S_0, and hot transitions are resonant with S_3 upon photoexcitation.

The experimental spectra at 355 nm from Refs. 13 and 17 are noticeably broader at the low eBE side, as compared to that from Ref. 14 (see Figure S7b). Although in all the cases, the experiments have been conducted at room temperature, the internal energy of anions extracted from an ion source before laser excitation can be different due to particular extraction and ion-thermalization processes. For example, ions are expected to be somewhat hotter, if they are directly exposed to a laser pulse in a single time-of-flight set-up,[13] thus explaining the difference in the photoelectron spectra. Furthermore, the observed broadening at the low eBE side is larger than that originating from hot transitions directly to the continuum (Figure S7b). This indicates that resonant PD may occur even at the excitation wavelength with the lowest total absorption cross-section, at which PD has previously been thought to be free of any impact of higher-lying resonance states.[13] At 355 and 350 nm, the ESR state is revealed at slightly shifted effective binding energies that are *lower* than 2.73 eV, since extra vibrational energy is borrowed from the ground-state nuclear dynamics (red spectra in Figures 4a and 4b of the main text). This consistently results in shifted to higher energies eKE^R distributions with respect to those of eKE^D shown in red and green, respectively, in Figures 4e and 4f.

We also note that the photoelectron distributions measured at a high photon energy at the edge of the $S_0 \rightarrow$ESR absorption band are additionally blurred, as compared to those calculated based on the statistical population of vibrational levels in the ESR state at the corresponding microcanonical temperature, which is $\sim$450 K for the room-temperature ground-state HBDI$^-$ anions excited at 315 nm. The high-energy photoexcitation populates excited vibrational levels of Franck-Condon active modes in ESR, thus resulting in many other low-intensity ESR$\rightarrow D_0$ transitions that are not fully accounted for in the statistical calculations (dashed red arrows in Figure 3a of the main text). A higher density of vibronic transitions within the energy domain between the direct and resonant parts at 315 nm is illustarted in Figure S10, where the resonant part is calculated based on both the statistical and initial (Franck-Condon) populations of vibrational levels in the ESR state. As a result, the 315 nm experimental photoelectron spectra (black and orange lines and shaded gray), which are defined by the direct and significantly shifted resonant parts, are completely unresolved, as opposed to the statistically calculated combined spectrum (purple line).

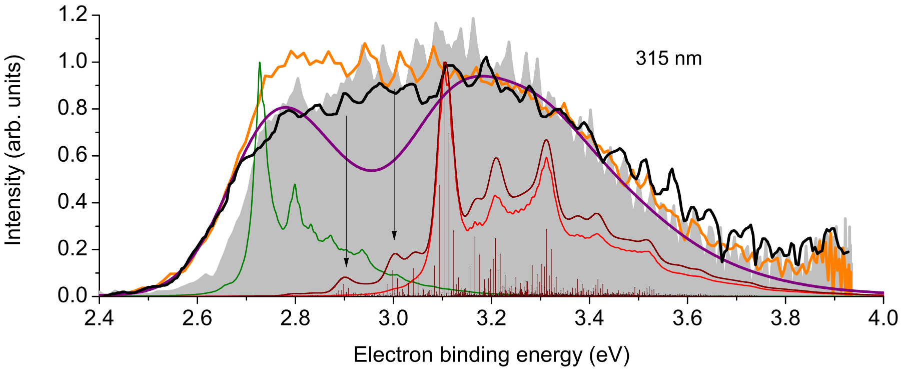

Figure S10: The 315 nm experimental (shaded gray (this work), orange line,[5] and black line[17]) and theoretical photoelectron spectra of HBDI$^-$. Shown are the statistically calculated direct (green line, 300 K) and resonant (red line, 400 K) parts, as well as the combined and convoluted theoretical spectrum (purple line). The resonant part calculated based on the initial Franck-Condon population of vibrational levels in the ESR state is shown as the stick spectrum as well as the stick spectrum convoluted with a Gaussian function with a HWHM of 13 meV (brown line). Note a higher density of the resonant transitions within the energy domain between the statistically calculated direct and resonant parts, which originate from initially populated excited vibrational levels.

10 Active modes in direct PD from DMHBDI$^-$ and DFHBDI$^-$

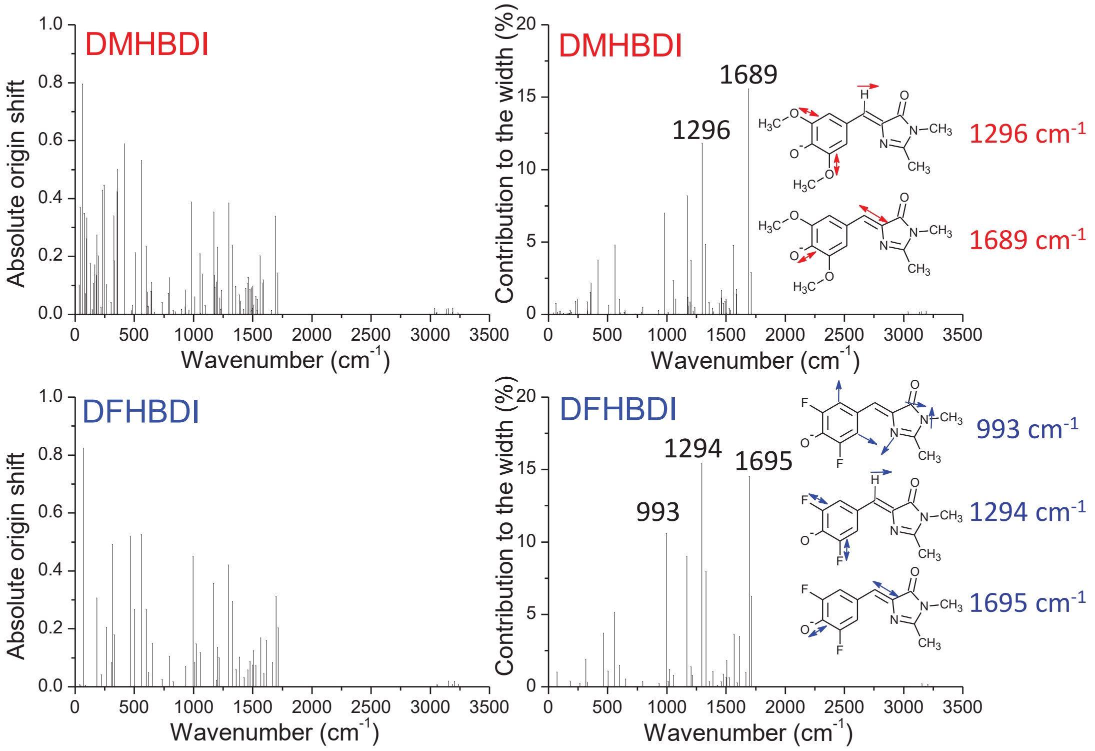

Figure S11: Franck-Condon active modes in direct PD from DMHBDI$^-$ and DFHBDI$^-$. Shown are absolute values of the S_0/D_0 dimensionless origin shifts along each normal mode (left) and contributions from each normal mode to the overall spectral width calculated as a Gaussian dispersion at 300 K (right). Harmonic MP2/(aug)-cc-pVTZ frequencies calculated in the ground electronic state of the anions are used for the analysis. The origin shifts of DMHBDI$^-$ are locally estimated based on the MRMP2/CASSCF(11,11)/(aug)-cc-pVTZ gradient in D_0 calculated at the MP2/(aug)-cc-pVTZ equilibrium geometry of the ground-state anion. This is done to avoid a mistreatment of the large-amplitude anharmonic torsional mode that is related to the sp^3–sp^2 change in hybridization of the oxygen atoms of the methoxy groups induced by electron detachment (Figure S13). When treated beyond the harmonic approximation, this low-frequency mode (79 cm^{-1}) will provide additional blurring of the spectral shape. All other modes are well described in the harmonic approximation. Their calculated origin shifts (less than $\sqrt{2}$) indicate that the 0–0 transitions have the maximum Franck-Condon strengths in both DMHBDI$^-$ and DFHBDI$^-$.

11 Impact of the substituents

The difluoro- and dimethoxy-substituents at the *ortho* positions in the phenoxide group of $HBDI^-$ both exhibit π electron-donating ($+M$) and σ electron-withdrawing ($-I$) effects. Their overall orienting character is, however, different. The fluoro-substituent shows an overall $-I$ character, whereas the methoxy one has a $+M$ character. The overall orienting effect results in stabilization or destabilization of the substituted anions against electron detachment; however, excitation energies in the anions are sensitive to the individual $+M$ or $-I$ effects, depending on character and symmetry of orbitals involved in transitions. Direct mixing of π (A$''$) or σ (A$'$) orbitals of the substituents with those of HBDI is only allowed for orbitals of the same type of symmetry.

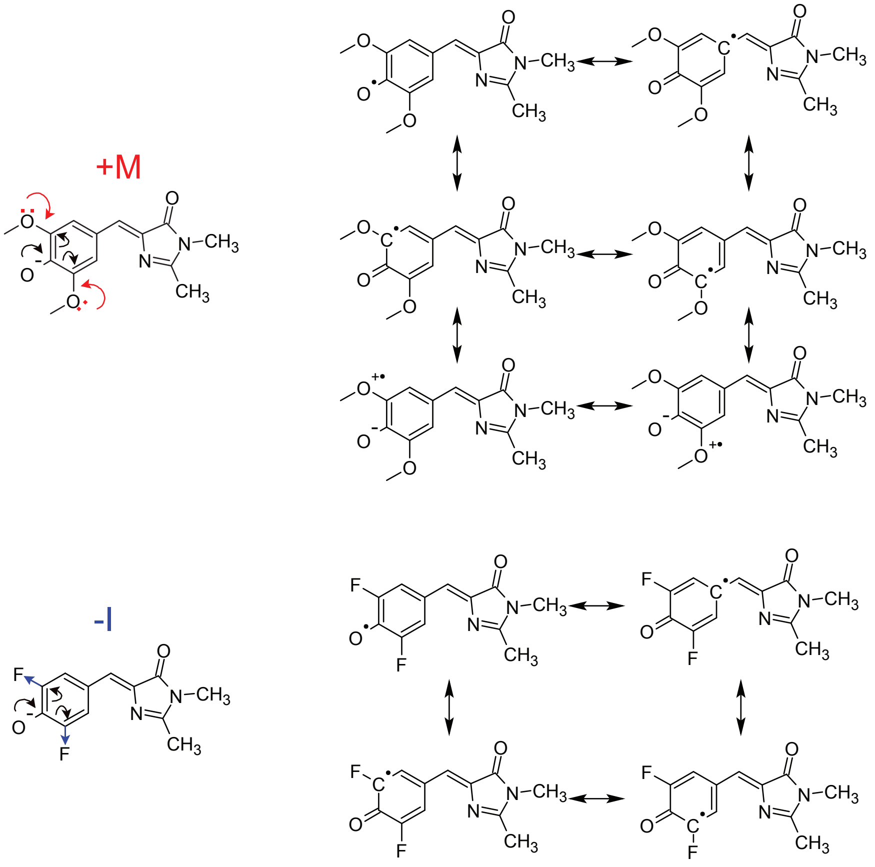

Figure S12: Impact of the dimethoxy- and difluoro-substituents. Shown are the overall $+M$ destabilizing and $-I$ stabilizing effects for the ground-state energies of the anions (left), as well as some of the resonance structures of the ground-state radicals (right). Note that two of the resonance structures of the methoxy substituted radical are stable captodative radicals.

The stabilization of the DMHBDI radical through the captodative effect involves the $sp^3 \rightarrow sp^2$ change in hybridization of the oxygen atoms of the methoxy groups induced by electron detachment, which enhances the interaction of the decoupled π-orbital of the substituents with the π conjugated system. The equilibrium geometry of the radical becomes nearly planar (see Figure S13).

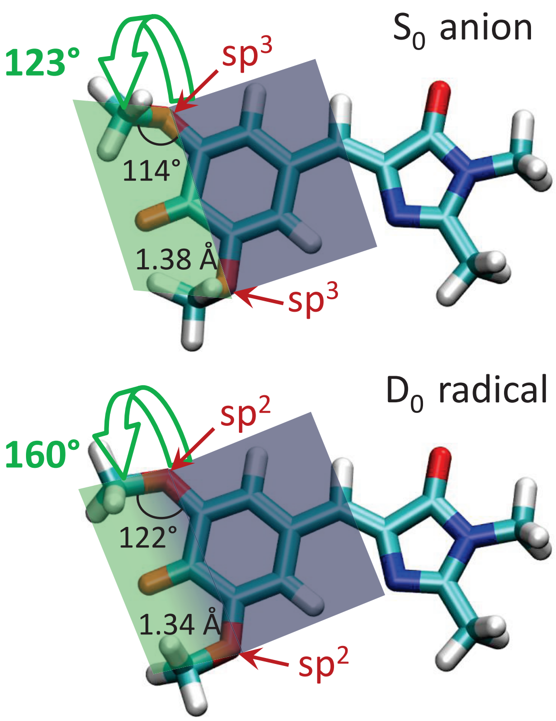

Figure S13: PBE0/(aug)-cc-pVTZ equilibrium geometries of the DMHBDI anion and the DMHBDI radical. Note the changes in hybridization of the oxygen atoms of the methoxy groups upon electron detachment. The captodative effect stabilizes the radical by extending the π conjugation. The resonance structures of the DMHBDI radical are shown in Figure S12.

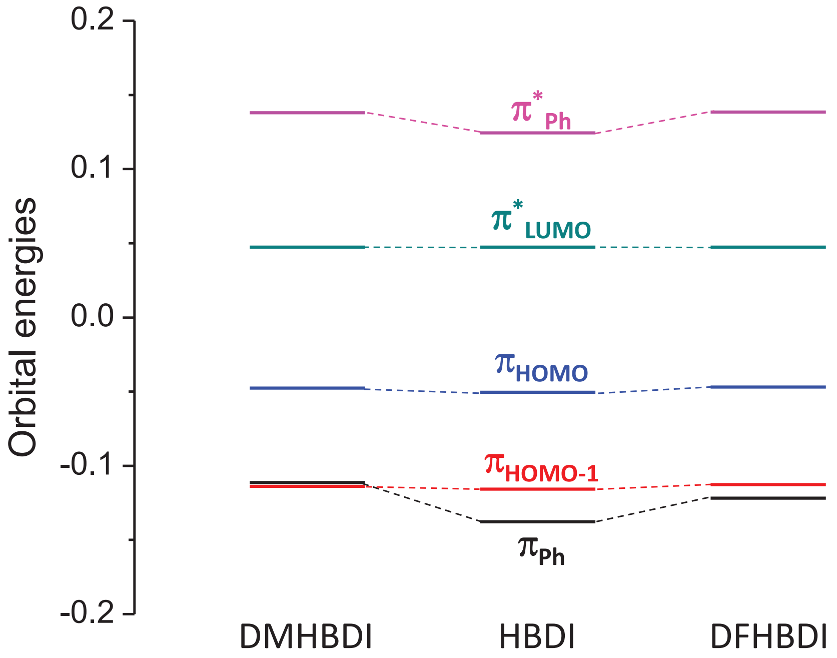

Figure S14: Destabilizing π electron donating effect ($+M$) of the substituents on energies of the active π orbitals. Shown are the energies defined as the diagonal values of a DFT/PBE0-based one-electron Fock matrix computed in the basis of the SA(7)-CASSCF(18,15) orbitals. The orbital energies of DMHBDI$^-$ and DFHBDI$^-$ are shifted by 0.007 and 0.015 a.u., respectively, to match the energy of the lowest unoccupied semi-canonical SA(7)-CASSCF(16,14) orbital of HBDI$^-$. Since this π^*_{LUMO} orbital does not directly mix with the π-orbitals of the substituents, the shift enables to eliminate all other effects, including a larger basis set, a larger number of electrons, and indirect inductive ($-I$) effects induced by the substituents. Note that the π orbitals localized on the phenoxide group, π_{Ph} and π^*_{Ph}, are significantly destabilized by the $+M$ effect, whereas π_{HOMO-1}, π_{HOMO}, and π^*_{LUMO} that define the three orbitals of the allyl bridge moiety are almost unaffected. The active π orbitals are shown in Figure S1.

12 XYZ coordinates of the optimized structures in Å

Table S4: Ground-state anions.

**HBDI anion**	MP2/(aug)-cc-pVTZ			**DFHBDI anion**	MP2/(aug)-cc-pVTZ		
	$E_{total} = -722.5299500$ a.u.; all real frequencies				$E_{total} = -920.8088746$ a.u.; all real frequencies		
C	1.6108486626	-1.9253454742	0.6879438455	C	1.6285829504	-1.9229115077	0.6764631573
N	2.6868281637	-1.8525549439	-0.0571747531	N	2.7020764585	-1.8565711227	-0.0709674636
N	0.8838312819	-3.0787405999	0.4808752720	N	0.8937065918	-3.0737785625	0.4767608920
C	1.5415454244	-3.8469259203	-0.4975145119	C	1.5433601006	-3.8483347945	-0.4998211246
O	1.1318511259	-4.9457934451	-0.9030562317	O	1.1318696731	-4.9446330146	-0.9024296747
C	2.6961615090	-3.0236477565	-0.8175930917	C	2.7035964009	-3.0302075697	-0.8273124524
C	3.6366774069	-3.4092481394	-1.7578565460	C	3.6380029914	-3.4207552146	-1.7662636934
H	3.4057076432	-4.3732424756	-2.2081760641	H	3.4088762995	-4.3839063462	-2.2174299550
C	4.8058132215	-2.7804759527	-2.2136373503	C	4.8085916007	-2.7853175175	-2.2187868823
C	5.2754137769	-1.5177158272	-1.7465961058	C	5.2627543170	-1.5258524631	-1.7424524152
H	4.6944163242	-1.0071679812	-0.9904808013	H	4.6977721314	-0.9986422531	-0.9873887155
C	5.5889975063	-3.4318655034	-3.2091782109	C	5.5911558902	-3.4336367980	-3.2119116981
H	5.2456174918	-4.3947484716	-3.5761178474	H	5.2839978177	-4.3953325585	-3.6047831336
C	6.4279045922	-0.9647352992	-2.2388661030	C	6.4139030271	-0.9872411263	-2.2429484977
H	6.7755206870	-0.0042268990	-1.8765517322	C	6.7367736195	-2.8573207517	-3.6840834279
C	6.7426547546	-2.8847237418	-3.7060767326	C	7.2635855940	-1.5844887275	-3.2526115400
H	7.3223367140	-3.3987134802	-4.4634354178	O	8.3150111139	-1.0638503649	-3.6912971609
C	7.2434293828	-1.6059023128	-3.2516427896	C	-0.3280733691	-3.4733948832	1.1189947252
O	8.3020135302	-1.0892122350	-3.6981685743	H	-0.5965689400	-4.4393808622	0.6948670912
C	-0.3373652092	-3.4885776822	1.1161217946	H	-1.1327685619	-2.7626580872	0.9279108686
H	-0.5972914893	-4.4543955575	0.6858313665	C	1.2070739974	-0.8868649352	1.6533918034
H	-1.1466213075	-2.7824431456	0.9255621714	H	1.1509156073	-1.2900139474	2.6665503648
C	1.1816499636	-0.8942875093	1.6672869174	H	0.2241299060	-0.4799534586	1.4075961581
H	1.1246004055	-1.2989157406	2.6801299838	H	1.9370241134	-0.0826163589	1.6335252740
H	0.1978164082	-0.4889077960	1.4211834240	H	-0.1987677382	-3.5793077270	2.1966119343
H	1.9087727551	-0.0871425392	1.6513321494	F	6.8258397958	0.2142669774	-1.7728257126
H	-0.2127094929	-3.5989628299	2.1941351211	F	7.4535604906	-3.5103903602	-4.6318596938
**DMHBDI anion**	MP2/(aug)-cc-pVTZ			**DMHBDI anion**	PBE0/(aug)-cc-pVTZ		
	$E_{total} = -951.2004318$ a.u.; all real frequencies				$E_{total} = -952.2260842$ a.u.; all real frequencies		
C	1.6848189331	-1.9190308576	0.6672819621	C	1.6765171629	-1.9229254921	0.6665422340
N	2.7326723363	-1.8208216749	-0.1122043682	N	2.7118332272	-1.8254270071	-0.0995047958
N	0.9875505601	-3.0983908114	0.5003730859	N	0.9826708680	-3.1032428661	0.4945006055
C	1.6372326279	-3.8586921940	-0.4874764127	C	1.6391330278	-3.8543734957	-0.4907642434
O	1.2550089192	-4.9744983563	-0.8654480686	O	1.2633480583	-4.9610608279	-0.8694345115
C	2.7560658696	-3.0010633117	-0.8580043719	C	2.7544234588	-2.9971892031	-0.8548741929
C	3.6741974427	-3.3673250769	-1.8222095844	C	3.6773252753	-3.3532001945	-1.8114368462
H	3.4661035696	-4.3441951298	-2.2542914365	H	3.4720750509	-4.3291153183	-2.2471324836
C	4.8087908264	-2.6983296055	-2.3214733702	C	4.8142973823	-2.6932540745	-2.3107545437
C	5.2401181362	-1.4218597419	-1.8764194917	C	5.2576957953	-1.4204856965	-1.8708376994
H	4.6802877620	-0.9009806716	-1.1125138783	H	4.6950497869	-0.8980854929	-1.1081649705
C	5.5874563573	-3.3453431659	-3.3158903132	C	5.5924919005	-3.3389230532	-3.3035322503
H	5.2967887240	-4.3261748906	-3.6758115728	H	5.2921293849	-4.3181137724	-3.6636002766
C	6.3584922437	-0.8319383243	-2.4144133306	C	6.3711080482	-0.8358556051	-2.3977487686
C	6.7062510990	-2.7570257882	-3.8548109707	C	6.7082783745	-2.7682488943	-3.8409662502
C	7.1545457413	-1.4328210937	-3.4727169011	C	7.1617437214	-1.4465252098	-3.4545987492
O	8.1347378121	-0.8495551229	-4.0153051778	O	8.1349907291	-0.8755089773	-3.9927375354
C	-0.1992031498	-3.5361885116	1.1815962619	C	-0.1998151927	-3.5489323520	1.1630048178
H	-0.4434401467	-4.5158414200	0.7742991485	H	-0.4370199823	-4.5315801170	0.7520702874
H	-1.0352959220	-2.8578822608	1.0076803385	H	-1.0472870361	-2.8789538600	0.9881913457
C	1.2540178302	-0.8886540177	1.6463098216	C	1.2436686549	-0.8945944867	1.6434484047
H	1.2419931145	-1.2822331684	2.6648196294	H	1.2286295788	-1.2861292604	2.6660122582
H	0.2502194252	-0.5198806760	1.4255592818	H	0.2364911410	-0.5249205140	1.4243658238
H	1.9534899391	-0.0589575763	1.5964093623	H	1.9416899843	-0.0611233598	1.5962208882
H	-0.0354586616	-3.6262116331	2.2560956501	H	-0.0493698388	-3.6443582160	2.2427441155
O	7.3682327075	-3.4256121903	-4.8663263246	O	7.3551909536	-3.4337665891	-4.8563402605
C	8.7322024797	-3.6901816483	-4.5464928171	C	8.7198939381	-3.7008156358	-4.6117623132
H	9.1540078332	-4.2022773884	-5.4087970355	H	9.1023358214	-4.2083657102	-5.4993652890
H	9.2672897433	-2.7612967892	-4.3581964307	H	9.2757570188	-2.7768751456	-4.4428744830
H	8.7920949260	-4.3444334463	-3.6716484286	H	8.8352529413	-4.3665841708	-3.7460474063
O	6.6859972852	0.4388455264	-1.9861101321	O	6.6947934664	0.4328155767	-1.9791843554
C	7.9833298605	0.5024971288	-1.3991651384	C	7.9861970248	0.5629384838	-1.4242864519
H	8.7423780899	0.1863328216	-2.1123595706	H	8.7537145132	0.2662929354	-2.1413339144
H	8.1397685514	1.5415393107	-1.1170601218	H	8.1088279751	1.6159128074	-1.1638307042
H	8.0200739144	-0.1248525572	-0.5036277789	H	8.0769772879	-0.0399108163	-0.5109694925

Table S5: Ground-state radicals.

**HBDI radical**	MRMP2/CASSCF(11,11)/(aug)-cc-pVTZ			**DFHBDI radical**	MRMP2/CASSCF(11,11)/(aug)-cc-pVTZ		
	$E_{total} = -722.4682163$ a.u.				$E_{total} = -920.7396526$ a.u.		
	$E_{relaxation} = 0.09$ eV				$E_{relaxation} = 0.11$ eV		
C	1.6293273141	-1.9290441100	0.6719641715	C	1.6501086857	-1.9218045900	0.6614897726
N	2.7145846615	-1.8637022443	-0.0849117830	N	2.7325925745	-1.8669443257	-0.1002184452
N	0.8822236063	-3.0755372541	0.4841784351	N	0.8943355411	-3.0645868508	0.4823196265
C	1.5229733234	-3.8495462848	-0.4856171013	C	1.5251320383	-3.8477347876	-0.4862434345
O	1.1462428398	-4.9382290179	-0.9088133615	O	1.1418596220	-4.9354491081	-0.9041499014
C	2.7117878717	-3.0289654285	-0.8326830183	C	2.7188233930	-3.0358595978	-0.8424705780
C	3.6346781883	-3.4468729709	-1.7807866557	C	3.6355311713	-3.4608531802	-1.7906741277
H	3.4019021958	-4.4088090708	-2.2283638945	H	3.4028818681	-4.4222936812	-2.2381037613
C	4.8142162553	-2.7939990359	-2.2286734982	C	4.8166325552	-2.8051527408	-2.2378153183
C	5.2505654581	-1.5278754911	-1.7351344240	C	5.2375969528	-1.5422878582	-1.7349754755
H	4.6529824746	-1.0371609457	-0.9797266667	H	4.6517712536	-1.0379596561	-0.9795121116
C	5.6051917419	-3.4541191969	-3.2353301417	C	5.6036415861	-3.4660410230	-3.2420012699
H	5.2664416290	-4.4151767741	-3.6043544005	H	5.2953415456	-4.4267123045	-3.6332346499
C	6.3935645656	-0.9514879739	-2.2052502456	C	6.3781987537	-0.9759095338	-2.2096304217
H	6.7346910584	0.0086236882	-1.8385381694	C	6.7461827206	-2.8805611612	-3.7057539015
C	6.7549863956	-2.8918317813	-3.7195009556	C	7.2373294206	-1.5848636639	-3.2334253686
H	7.3584101937	-3.3772222603	-4.4757245208	O	8.2766213870	-1.0528480883	-3.6556185816
C	7.2147523264	-1.5987337987	-3.2258506655	C	-0.3343853394	-3.4310161113	1.1513033667
O	8.2659312223	-1.0673870965	-3.6573478404	H	-0.6374181317	-4.3972976041	0.7523448706
C	-0.3465066585	-3.4541494866	1.1452501060	H	-1.1212332380	-2.7029058827	0.9553185336
H	-0.6404015236	-4.4205192090	0.7396196211	C	1.2528032163	-0.8706376595	1.6307811981
H	-1.1381209563	-2.7309841427	0.9496141832	H	1.1992786448	-1.2754157731	2.6428914022
C	1.2207023011	-0.8850690290	1.6446362030	H	0.2709416618	-0.4640093385	1.3824467744
H	1.1654418501	-1.2943715244	2.6548938923	H	1.9883808091	-0.0713846275	1.6035586314
H	0.2372879432	-0.4832302473	1.3943937651	H	-0.1846808789	-3.5216933976	2.2269672398
H	1.9513917066	-0.0811676300	1.6239314595	F	6.7891736279	0.2082134557	-1.7501209374
H	-0.2017309822	-3.5495479124	2.2212786216	F	7.4874530932	-3.4834111070	-4.6388628342

**DMHBDI radical**	PBE0/(aug)-cc-pVTZ		
	$E_{total} = -952.1284926$ a.u.; all real frequencies		
	$E_{relaxation} = 0.23$ eV		
C	1.6682526578	-1.9221474045	0.6406463446
N	2.7303465602	-1.8445271915	-0.1019145187
N	0.9364692957	-3.0735341325	0.4612983491
C	1.5820698684	-3.8427978260	-0.5041425064
O	1.2148363617	-4.9223313759	-0.9090241973
C	2.7582787563	-3.0044985155	-0.8542490932
C	3.6620014852	-3.4063292194	-1.7851019724
H	3.4387106526	-4.3729628866	-2.2273096399
C	4.8385281390	-2.7502770795	-2.2569069257
C	5.2667921026	-1.4982385233	-1.7673995934
H	4.6912730520	-0.9958096386	-1.0031457435
C	5.6026066274	-3.4168679220	-3.2359452898
H	5.2882128207	-4.3874686261	-3.5995823912
C	6.4027066748	-0.9119034258	-2.2496719405
C	6.7458797226	-2.8669266512	-3.7477518340
C	7.1796695310	-1.5284069834	-3.3331985889
O	8.1070507464	-0.9351320922	-3.8962778941
C	-0.2801164301	-3.4695961178	1.1168430160
H	-0.5521732169	-4.4467136779	0.7189739890
H	-1.0904940248	-2.7669835040	0.9132893977
C	1.2495638711	-0.8839941329	1.6063658136
H	1.2031164283	-1.2886052845	2.6210296248
H	0.2541908761	-0.5021717748	1.3641703694
H	1.9643784992	-0.0656537918	1.5788711411
H	-0.1425110935	-3.5528314468	2.1965357475
O	7.3803891301	-3.5625533688	-4.6981918170
C	8.7502327699	-3.3419132156	-5.0161414999
H	9.0618172164	-4.2405134484	-5.5467827485
H	8.8758745749	-2.4648310311	-5.6467578834
H	9.3512541412	-3.2201742370	-4.1145246841
O	6.7160534013	0.2956345681	-1.7623425743
C	8.0487672752	0.7939358237	-1.7788457780
H	8.3038871150	1.2168585249	-2.7477569108
H	8.0702105274	1.5653849915	-1.0100011071
H	8.7676757581	0.0119137638	-1.5315254161

References

(1) Paige, J. S.; Wu, K. Y.; Jaffrey, S. R. *Science* **2011**, *333*, 642.

(2) Garcia, G. A.; Nahon, L.; Powis, I. *Rev. Sci. Instrum.* **2004**, *75*, 4989.

(3) McKay, A. R.; Sanz, M. E.; Mooney, C. R. S.; Minns, R. S.; Gill, E. M.; Fielding, H. H. *Rev. Sci. Instr.* **2010**, *81*, 123101.

(4) Mooney, C. R. S.; Sanz, M. E.; McKay, A. R.; Fitzmaurice, R. J.; Aliev, A. E.; Caddick, S.; Fielding, H. H. *J. Phys. Chem. A* **2012**, *116*, 7943.

(5) Mooney, C. R. S.; Parkes, M. A.; Zhang, L.; Hailes, H. C.; Simperler, A.; Bearpark, M. J.; Fielding, H. H. *J. Chem. Phys.* **2014**, *140*, 205103.

(6) Mooney, C. R. S.; Parkes, M. A.; Iskra, A.; Fielding, H. H. *Angew. Chem. Int. Ed.* **2015**, *54*, 5646.

(7) Granovsky, A. A. *J. Chem. Phys.* **2011**, *134*, 214113.

(8) Granovsky, A. A. Firefly, version 8.1, http://classic.chem.msu.su/gran/firefly/index.html.

(9) Schiedt, J.; Weinkauf, R. *J. Chem. Phys.* **1999**, *110*, 304.

(10) Bochenkova, A. V.; Andersen, L. H. *Faraday Discuss.* **2013**, *163*, 297.

(11) Lax, M. *J. Chem. Phys.* **1952**, *20*, 1752.

(12) Yurenev, P. V.; Kretov, M. K.; Scherbinin, A. V.; Stepanov, N. F. *J. Phys. Chem. A* **2010**, *114*, 12804.

(13) Toker, Y.; Rahbek, D. B.; Klærke, B.; Bochenkova, A. V.; Andersen, L. H. *Phys. Rev. Lett.* **2012**, *109*, 128101.

(14) Deng, S. H. M.; Kong, X.-Y.; Zhang, G.; Yang, Y.; Zheng, W.-J.; Sun, Z.-R.; Zhang, D.-Q.; Wang, X.-B. *J. Phys. Chem. Lett.* **2014**, *5*, 2155.

(15) Bochenkova, A. V.; Klærke, B.; Rahbek, D. B.; Rajput, J.; Toker, Y.; Andersen, L. H. *Angew. Chem. Int. Ed.* **2014**, *53*, 9797.

(16) Almasian, M.; Grzetic, J.; Berden, G.; Bakker, B.; Buma, W. J.; Oomens, J. *Int. J. Mass. Spect.* **2012**, *330–332*, 118.

(17) West, C. W.; Bull, J. N.; Hudson, A. S.; Cobb, S. L.; Verlet, J. R. R. *J. Phys. Chem. B* **2015**, *119*, 3982.
